# Pharmacological Mechanisms of Tinglizi against Chronic Heart Failure Determined by Network Pharmacology and Molecular Docking

**DOI:** 10.1155/2022/2152399

**Published:** 2022-01-06

**Authors:** Liangtao Luo, Haowen Wang, Guowei Huang, Lu Zhang, Xiuwei Li, Chongyang Ma, Xing Wang

**Affiliations:** ^1^School of Traditional Chinese Medicine, Capital Medical University, Beijing 10069, China; ^2^School of Medical Humanities, Capital Medical University, Beijing 10069, China

## Abstract

**Objective:**

Tinglizi has been extensively used to treat chronic heart failure (CHF) in modern times, but the material basis and pharmacological mechanisms are still unclear. To explore the material basis and corresponding potential targets and to elucidate the mechanism of Tinglizi, network pharmacology and molecular docking methods were utilized.

**Methods:**

The main chemical compounds and potential targets of Tinglizi were collected from the pharmacological database analysis platform (TCMSP). The corresponding genes of related action targets were queried through gene cards and UniProt database. The corresponding genes of CHF-related targets were searched through Disgenet database, and the intersection targets were obtained by drawing Venn map with the target genes related to pharmacodynamic components. Then, drug targets and disease targets were intersected and put into STRING database to establish a protein interaction network. The “active ingredient-CHF target” network was constructed with Cytoscape 3.8.2. Finally, Gene Ontology (GO) Enrichment and Kyoto Encyclopedia of Genes and Genomes (KEGG) pathway enrichment of intersection targets were analyzed using metascape. With the aid of SYBYL software, the key active ingredients and core targets were docked at molecular level, and the results were visualized by PyMOL software. Molecular docking was carried out to investigate interactions between active compounds and potential targets.

**Results:**

A total of 12 active components in Tinglizi were chosen from the TCMSP database, and 193 corresponding targets were predicted. Twenty-nine potential targets of Tinglizi on CHF were obtained, of which nine were the core targets of this study. Twenty GO items were obtained by GO function enrichment analysis (*P* < 0.05), and 10 signal pathways were screened by KEGG pathway enrichment analysis (*P* < 0.05), which is closely related to the treatment of CHF by Tinglizi. The constructed drug compound composition action target disease network shows that quercetin, kaempferol, and other active compounds play a key role in the whole network. The results of molecular docking showed that all the key active ingredients, such as quercetin and isorhamnetin, were able to successfully dock with ADRB2 and HMOX1 with a total score above 5.0, suggesting that these key components have a strong binding force with the targets.

**Conclusion:**

Through network pharmacology and molecular docking technology, we found that the main components of Tinglizi in the treatment of CHF are quercetin, kaempferol, *β*-sitosterol, isorhamnetin, and so on. The action targets are beta 2-adrenergic receptor (ADRB2), heme oxygenase 1 (HMOX1), and so on. The main pathways are advanced glycation end products/receptor for advanced glycation end products (AGE-RAGE) signaling pathway in diabetic complications, hypoxia-inducible factor (HIF-1) signaling pathway, estrogen signaling pathway, and so on. They play an integrated role in the treatment of CHF.

## 1. Introduction

Heart failure (HF) is a complex clinical syndrome that results from any structural or functional impairment of ventricular filling or ejection of blood. According to the time and progression of heart failure, it can be divided into CHF and acute heart failure (AHF). The cardinal manifestations of HF are dyspnoea and fatigue, which may limit exercise tolerance, and fluid retention, which may lead to pulmonary and/or splanchnic congestion and/or peripheral oedema. Some patients have exercise intolerance but little evidence of fluid retention, whereas others complain primarily of oedema, dyspnoea, or fatigue [1]. Most patients with AHF are partially relieved after hospitalization and then develop CHF. Patients with CHF often need hospitalization because of acute exacerbation of various origins.

The latest epidemiological survey of HF in China shows that the prevalence rate of residents over 35 is 1.3%, and it is estimated that currently there are about 8.9 million patients with HF in China [2]. The treatment goal of CHF is to relieve clinical symptoms and improve quality of life, to prevent or reverse cardiac remodeling, to reduce rehospitalization, and to decrease mortality [3]. At present, the therapeutic drugs are diuretics, renin angiotensin system inhibitors, and angiotensin receptor-neprilysin inhibitor. However, after the treatment of drugs recommended in the guidelines, some symptoms remain to be alleviated. Also, the long-term use of relevant drugs will cause adverse reactions, such as hypotension, bradycardia, deterioration of renal function, and hyperkalemia [3]. Traditional Chinese medicine has certain advantages in improving the symptoms and signs of CHF patients. In recent years, the clinical evidence of traditional Chinese medicine (TCM) applied in the treatment of CHF has increased, and TCM has been included in guidelines for CHF in China [3]. The number of Chinese patent medicines that can be used to treat CHF is increasing year by year. Among them, Qiliqiangxin capsule and Qishen Yiqi dropping pill have a significant curative effect and sufficient clinical research evidence [4, 5]. Over the past 20 years, after screening, 210 kinds of Chinese herbal medicine are involved in the treatment of CHF. Among them, Tinglizi (pepperweed seed, *Semen Descrurainiae seu Lepidii*) has been quite frequently used, ranking the sixth [6].

Tinglizi is sourced from the ripe seeds of *Descurainia Sophia (L.)* Webb. Ex Prantl. and *Lepidium apetalum* Willd [7]. Tinglizi is often used to treat CHF, cough and dyspnOea, ascites, tumor, and other diseases [8]. Pharmacological research of Chinese Materia Medica shows that Tinglizi has the functions of diuresis, strengthening muscles, inhibiting ventricular remodeling, and protecting cardiomyocytes, so it can significantly alleviate the clinical symptoms of CHF [8]. However, the current research mainly focuses on the monomer components of Tinglizi, while its molecular mechanism and the interaction between its various components need to be further clarified.

Based on systemic biology and pharmacology, network pharmacology provides a new “multitarget, multichannel, and multilink” network model. It offers a new research paradigm for the transformation of TCM from empirical medicine to evidence-based medicine. Network pharmacology has become an indispensable method to explore the potential mechanism of TCM [9, 10].

Molecular docking is one of the most commonly used methods in drug design. It can predict the binding conformation between small molecule ligands and target binding sites. In this study, by searching the effective components and target proteins and constructing the target network, the core targets in the network were determined, and the biological function and pathway enrichment analysis were carried out. The molecular docking between the effective components and core targets of Tinglizi was verified by molecular docking technology, in order to explore the possible pharmacological mechanism of Tinglizi in the treatment of CHF at the molecular level.

## 2. Materials and Methods

### 2.1. Active Compounds' Collection

The TCMSP (https://lsp.nwu.edu.cn/tcmsp.php) [11] is generally used as a platform for systems pharmacology and a compound ingredient database. The main active compounds were searched in TCMSP database. The threshold values of oral bioavailability (OB) were set as ≥30%, and drug-likeness (DL) was set as ≥0.18. Finally, effective components and targets were treated as the candidate active ingredients [12].

### 2.2. Screening of CHF-Related Targets

We searched the target genes related to CHF with the keyword “chronic heart failure” in Disgenet database (https://www.disgenet.org/) [13]. The human-related genes corresponding to the target of the effective components of Tinglizi were searched in UniProt database (https://www.uniprot.org/) [14]. We intersected the target genes related to CHF with the target genes corresponding to the active ingredient and presented it using Venn diagram to obtain the intersection target of active ingredient and disease.

### 2.3. Construction and Analysis of Protein–Protein Interaction Network (PPI)

STRING is a known database for predicting protein–protein interactions. Here, the intersection target of effective components of Tinglizi and CHF was entered into the STRING database (Search Tool for the Retrieval of Interacting Genes/Proteins, https://string-db.org/) [15], and the species was limited to humans. The PPI network of Tinglizi and its effective components for the treatment of CHF was constructed. We calculated the degree value of each node on PPI background, which was defined by the number of edges connected by one node. The greater the degree value is, the more important the node is.

### 2.4. Construction of “Active Ingredient Target” Network Diagram

The visual “active ingredient-CHF target” network was constructed with Cytoscape 3.8.2 software. The nodes in the network represent active components or CHF targets, and the edges between nodes represent the interaction between active components and targets.

### 2.5. Functional Enrichment Analysis of Key Targets

GO enrichment and KEGG pathway enrichment of target of Tinglizi in the treatment of CHF were analyzed using an R package, clusterProfiler. After screening, GO enrichment and KEGG pathway with *P* < 0.05 were chosen.

### 2.6. Component Target Molecular Docking

Molecular docking studies were performed by Sulflex-Dock module in the SYBYL X-1.2 software (Tripos, Inc., St. Louis, MO, USA). The crystal structure of protein was obtained from RCSB Protein Date Bank (http://www.rcsb.org/). Hydrogen atoms, charges were added, and missing amino acid residues were repaired to the target protein by SYBYL X-1.2 software. The protein model was first optimized in AMBER FF99 force field for 1000 iterations by steepest descent (SD) and then optimized to a convergence gradient as 0.05 kcal/(Å mol) by conjugated gradient (CG). The planar structures of active compounds were converting into 3D structure. Polar hydrogens and charges were added by Gasteiger–Hückel method, and energy optimization was implemented using the Tripos force-field for 1000 iterations. After completing each run of the docking, the ten best conformers achieved through docking were then sorted in a molecular spreadsheet. Their binding affinities were represented as -lgKd based on the surflex-dock scoring function (crash score, D-score, polar score, G-score, CScore, ChemSco, and PMF-score).

## 3. Results

### 3.1. Active Compounds of Tinglizi

After screening the TCMSP database, we selected 12 active components with good oral bioavailability and drug-likeness on the conditions of “OB ≥ 30%, DL ≥ 0.18” as shown in Table 1.

### 3.2. Prediction of Potential Targets of Tinglizi in the Treatment of CHF

**Figure 1:**
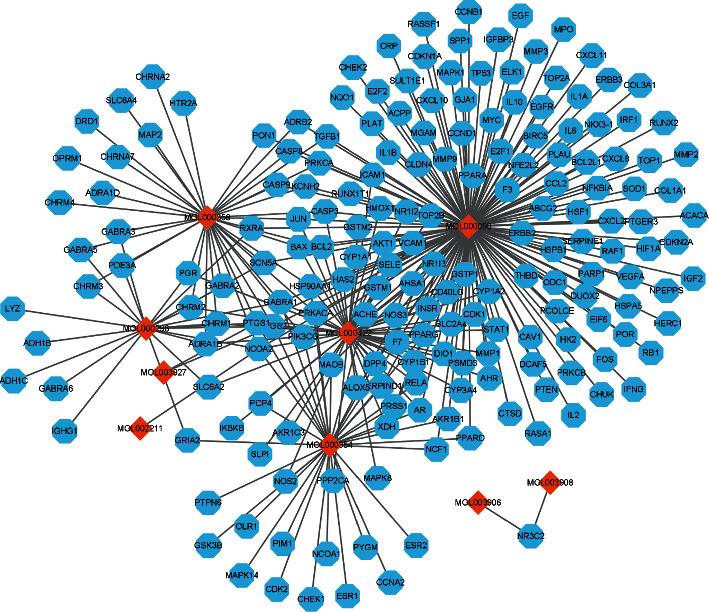
Active ingredient target overall network.

One hundred ninety-three Tinglizi-related action targets were retrieved from TCMSP database. Then, we converted 193 targets protein into gene names by GENECARD and UniProt database and constructed the overall network of “active ingredient targets” by Cytoscape 3.8.2 as shown in Figure 1.

### 3.3. Construction and Analysis of “Active Ingredient-CHF Targets” Network

**Figure 2:**
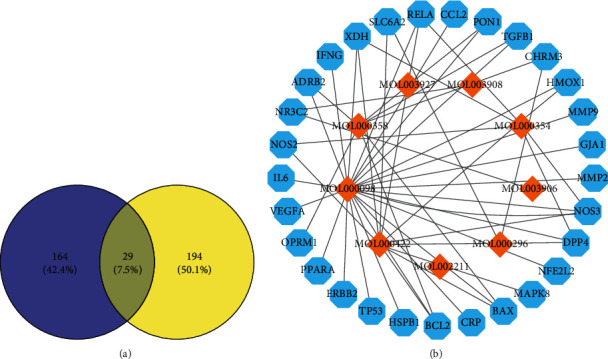
Screening common targets of Tinglizi and chronic heart failure. (a) The Venn diagram of Tinglizi targets and CHF targets. (b) Active ingredient-CHF targets network.

In the Disgenet database, we retrieved 223 CHF-related targets, took the intersections of the two, and drew 29 intersection targets in the Venn diagram, as shown in Figure 2(a). The 29 targets obtained from the intersections in Figure 2 are solute carrier family 6 member 2 (SLC6A2), recombinant nitric oxide synthase 2 (NOS2), dipeptidyl peptidase 4 (DPP4), recombinant nitric oxide synthase 3 (NOS3), RELA proto-oncogene (RELA), xanthine dehydrogenase (XDH), adrenoceptor beta 2 (ADRB2), opioid receptor Mu 1 (OPRM1), B-cell lymphoma-2 protein (BCL2), BCL2-associated X protein (BAX), transforming growth factor beta 1 (TGFB1), paraoxonase 1 (PON1), nuclear receptor subfamily 3 group C member 2 (NR3C2), mitogen-activated protein kinase 8 (MAPK8), heme oxygenase 1 (HMOX1), vascular endothelial growth factor A (VEGFA), matrix metallopeptidase 2 (MMP2), matrix metallopeptidase 9 (MMP9), IL-6, tumor protein P53 (TP53), Erb-B2 receptor tyrosine kinase 2 (ERBB2), gap junction protein alpha 1 (GJA1), C-C motif chemokine ligand 2 (CCL2), heat shock protein family B (small) member 1 (HSPB1), interferon gamma (IFNG), nuclear factor, erythroid 2-like 2 (NFE2L2), peroxisome proliferator activated receptor alpha (PPARA), and C-reactive protein (CRP). We draw the “active ingredient-CHF” network as shown in Figure 2(b).

Among them, the yellow diamonds represent the effective components of Tinglizi, and the blue hexagons represent the targets of related components in the treatment of CHF. There are 38 nodes and 50 edges in the network. The extent of interaction between the effective components and targets is expressed by degree. The degree value of key component mol000098 (quercetin) is 23, that of mol000422 (kaempferol) 10, that of mol000358 (beta-sitosterol) 7, and that of mol000354 (isorhamnetin) 5.

As is shown in Figure 2(b), Tinglizi mainly acts on 29 targets through nine effective components. Each component corresponds to one or more targets, which can reflect its characteristics of multicomponent, multitarget, and multichannel treatment of CHF.

### 3.4. Construction and Analysis of PPI Network of Intersection Target between Tinglizi and CHF

**Figure 3:**
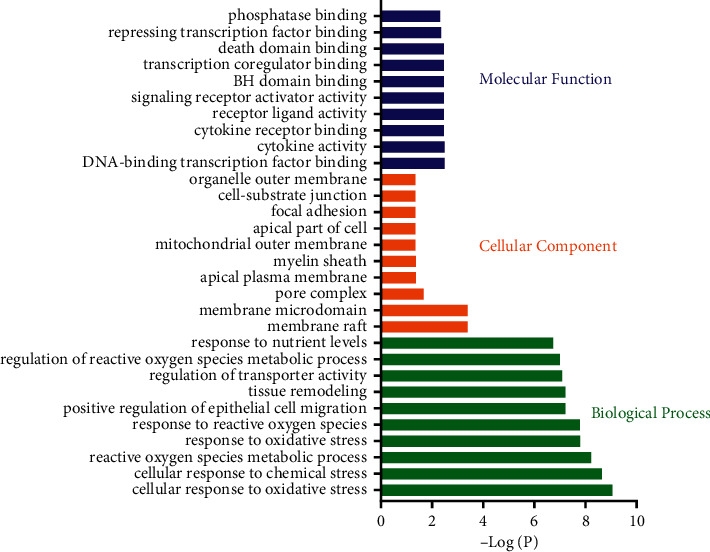
Gene oncology enrichment of common targets.

**Figure 4:**
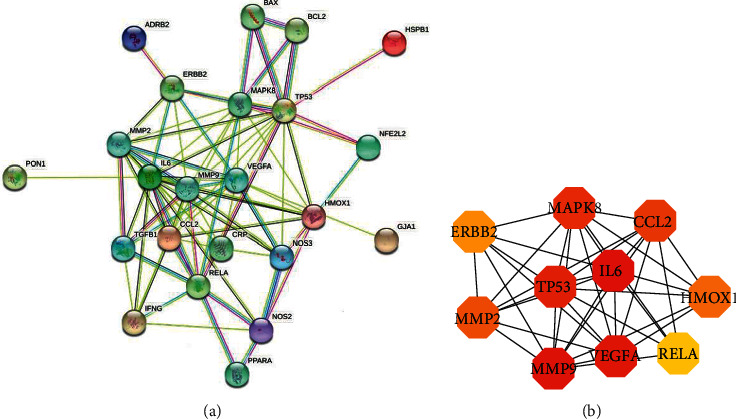
PPI network of Tinglizi in the treatment of CHF. (a) PPI network from String database. (b) Top ten targets analyzed by cytoHubba.

The PPI network was built in the STRING database, as shown in Figure 3. It contains 29 nodes and 78 edges (Figure 4(a)). Six of the nodes were free nodes and did not participate in protein –protein interaction. The network data was imported into Cytoscape 3.8.2 software for visual processing, and the nodes were protein nodes. In this paper, the top ten protein nodes (Table 2) in terms of degree were selected for mapping, as shown in Figure 4(b). The connections between these proteins are relatively dense, indicating that the interaction between these proteins is the closest during the treatment of CHF.

### 3.5. Go Enrichment Analysis

We used the clusterProfiler package to perform GO enrichment analysis on 29 gene targets. Only GO terms with *P* < 0.05 were considered as significant and top 10 terms were visualized. A total of 1323 items were obtained in the biological process analysis, mainly including cellular response to oxidative stress, cellular response to chemical stress, reactive oxygen species metabolic process, response to oxidative stress, response to reactive oxygen species, positive regulation of epithelial cell migration, tissue remodeling, regulation of transporter activity, regulation of reactive oxygen species metabolic process, and response to nutrient levels, as shown in Figure 3.

In the part of cellular component analysis, 12 items were obtained, mainly including membrane raft, membrane microdomain, pore complex, apical plasma membrane, myelin sheath, mitochondrial outer membrane, apical part of cell, focal adhesion, cell-substrate junction, and organelle outer membrane, as shown in Figure 3.

A total of 41 items were obtained in the molecular function analysis part, mainly including DNA-binding transcription factor binding, cytokine activity, cytokine receptor binding, receptor ligand activity, signaling receptor activator activity, BH domain binding, transcription coregulator binding, death domain binding, repressing transcription factor binding, and phosphatase binding, as shown in Figure 3.

### 3.6. KEGG Enrichment Analysis

**Figure 5:**
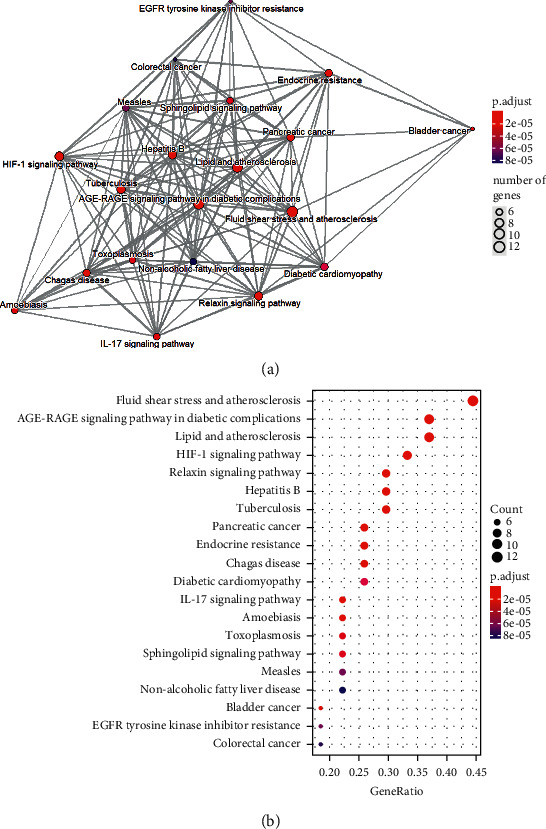
KEGG pathway enrichment analysis of common targets. (a) Emapplot of enriched pathways. (b) Bubble plot of enriched pathways.

Similarly, we used the R package to perform KEGG enrichment analysis on 29 gene targets. Only KEGG terms with *P* < 0.05 were considered as significant, and 104 relevant pathways were obtained. In Table 3, top 40 pathways were listed sorted by *P* values. As shown in Figures 5(a) and 5(b), top 20 KEGG pathways were visualized. It can be found that the pathways of Tinglizi for CHF may include fluid shear stress and atherosclerosis, AGE-RAGE signaling pathway in diabetic complications, HIF-1 signaling pathway, estrogen signaling pathway, and calcium signaling pathway.

### 3.7. Molecular Docking Verification of Core Compounds and Core Protein Targets

To further explore the interactions between the active ingredients of Tinglizi and key targets, molecular docking studies were performed between 12 active compounds and the selected potential targets. These targets were chosen for docking because of their higher degree and betweenness centrality value in PPI analysis and also superior performance in clustering analysis. The compound–target pairs with the score ≥4 are considered to have particular binding activity, and when the score ≥6, it indicates strong binding efficiency between the compound and the target. As shown in Supplementary Table 1, the key compound quercetin could bind to the ADRB2 protein via hydrogen bonds (via ASP1113, Asn1312, ALA1200, SER1204, and SER1207) and hydrophobic interactions (VAL1114, PHE1290, PHE1289, and PHE1193). Isorhamnetin could bind to the HMOX1 protein via hydrogen bonds (via SER630, CYS645, and VAL647) and hydrophobic interactions (TYR629, ALA646, and TRP850). The docking results show that multiple active ingredients of Tinglizi have a great binding efficiency with the key targets, which further indicates the characteristics of multicomponent, multitarget of Tinglizi against CHF.

## 4. Discussion

CHF is a major public health challenge in China. With the worsening population aging in China, the incidence of chronic diseases, such as coronary heart disease, hypertension, diabetes, and obesity, is showing an upward trend. The survival time of patients with heart disease has prolonged for the improvement of medical conditions. The prevalence rate of CHF in China has been increasing. TCM has a long history of clinical practice, and many Chinese herbal medicines have played good clinical effects in the treatment of CHF. With the increase of clinical evidence, TCM is more and more recognized. Tinglizi was first recorded in *Shennong's Book of Materia Medica*. Literature mining suggested that Tinglizi was one of the most commonly used Chinese herbal medicines for the treatment of CHF [16]. In order to further understand the potential mechanism, we used systematic pharmacological methods and molecular docking to explore the potential molecular mechanism of CHF.

The TCMSP database and Cytoscape 3.8.2 were used to construct the pharmacodynamic target component interaction network. Nine pharmacodynamic components were selected for the treatment of CHF, including quercetin, kaempferol, *β*-sitosterol, and isorhamnetin. They have effect on 29 targets. Quercetin, kaempferol, and isorhamnetin play a great role in the treatment of CHF. This study suggests that quercetin, kaempferol, and isorhamnetin play a significant role in the treatment of CHF.

The mechanism of quercetin in the treatment of CHF may be through the expression of various regulatory proteins, inhibition of MAPK pathway, inhibition of inflammatory response, inhibition of oxidative stress, and so on. Studies have shown that quercetin can downregulate the expression of NF-*κ*B protein to reduce ventricular hypertrophy in rats [17–19]. Quercetin could inhibit the proliferation of myocardial fibroblasts and improve ventricular remodeling. The mechanism may be to inhibit the activation of MAPK pathway through ROS [20]. Quercetin inhibited myocardial fibrosis and improved cardiac function by increasing mitochondrial energy metabolism and regulating mitochondrial fusion/fission and mitochondrial biosynthesis while inhibiting the inflammatory response and oxidative stress injury [21]. Kaempferol (KPF) protects cardiomyocytes from many aspects because of its significant inhibition of inflammatory response and oxidative stress [22]. Kaempferol can protect heart injury caused by hyperglycemia by anti-inflammatory and inhibiting oxidative stress, mechanically linked to inhibition of NF-*κ*B and Nrf-2 activation [23]. Kaempferol is a flavonoid compound with anti-inflammatory and antioxidant effects. KPF prevented Ang II-induced cardiac fibrosis and dysfunction, in mice challenged with subcutaneous injection of Ang II. KPF remarkably decreased inflammation and oxidative stress in Ang II-stimulated cardiac fibroblasts by modulating NF-*κ*B/mitogen-activated protein kinase and AMPK/Nrf2 pathways [24]. *β*-Sitosterol has anti-inflammatory effects. Sitosterol may inhibit renal and cardiac necrosis and apoptosis by limiting inflammatory response and oxidative stress [25]. Isorhamnetin has a protective effect on ischemia–reperfusion injury of isolated rat hearts [26]. ISO was found to reverse the enhanced TGF-*β* and collagen type I alpha 1 mRNA expression induced by AgII exposure, which causes cardiovascular remodeling in ventricular tissue [27]. Isorhamnetin treated myocardial-related diseases by regulating PI3K, Akt, and NF-*κ*B signaling pathway [28]. Components of Tinglizi, such as quercetin, kaempferol, *β*-sitosterol, and isorhamnetin, collectively resist inflammation and oxidative stress, protect cardiomyocytes, and inhibit ventricular remodeling to treat CHF.

PPI protein interaction diagram shows that the targets of Tinglizi in the treatment of CHF include IL-6, MMP9, VEGFA, MAPK8, HMOX1, and so on. There is a significant correlation between serum IL-6 level and cardiac function in patients with CHF. It may be used as an evaluation index for the severity and prognosis of elderly patients with CHF. Inhibiting the increase of IL-6 level plays an important role in the phased treatment of CHF [29, 30]. IL-6 is consistently upregulated in experimental models of cardiac injury and heart failure regardless of the underlying etiology and is expressed by cardiomyocytes, infiltrating mononuclear cells, and fibroblasts. IL-6 has been reported to exert both pro- and anti-inflammatory actions, stimulate fibroblast proliferation and ECM synthesis, and promote cardiomyocyte hypertrophy. MMP-9 is an important member of the MMPs family. It participates in the synthesis and degradation of extracellular matrix and the regulation of inflammatory mediators, leading to myocardial remodeling and promoting the occurrence and development of CHF. Increased activity of MMP-9 is found in the heart failure rat model and the myocardium of patients with heart failure, which affects the heart function of patients with CHF [31, 32]. The activity of MMP-9 increases in patients with dilated cardiomyopathy, and the expression of MMP-9 is significantly higher in patients with heart failure for any reason. According to NYHA classification, a study found that the more severe the HF, the higher the MMP-9 level [33]. VEGF is an important growth factor, which is involved in many processes of human growth and development, such as angiogenesis and osteogenesis, and plays an important role in maintaining vascular homeostasis and normal cardiac function [34]. The level of VEGFA can regulate the HIF-1/VEGFA signaling pathway and reverse hypoxia-induced myocardial injury by regulating the level of HMOX1 [35]. MAPK is a group of serine-threonine protein kinases that can be activated by different extracellular stimuli, such as cytokines, neurotransmitters, hormones, cell stress, and cell adhesion. It is an important transmitter of signals from the cell surface to the nucleus and regulates cell growth, differentiation, stress adaptation to the environment, inflammation response, and other important cellular physiological/pathological processes. MAPK pathway is one of the common intersection pathways of signal transduction pathways, such as cell proliferation, stress, inflammation, differentiation, functional synchronization, transformation, and apoptosis. MAPK8 may achieve the protective effect on cardiomyocytes by regulating the inflammatory response and apoptosis of cardiomyocytes. Furthermore, it is an important heart-rate-related gene [36].

GO biological process analysis showed that the targets of Tinglizi are mainly related to the regulation of cell proliferation, anti-inflammatory response, inhibition of cell oxidative stress, and regulation of cell apoptosis. It is suggested that the effective components of Tinglizi may play a role in CHF treatment through the above biological processes.

KEGG pathway enrichment analysis showed that pathway ko05418 (fluid shear stress and atherosclerosis) is an important way to treat CHF. Coronary artery disease is a serious cardiovascular disease, because atherosclerosis can hinder the flow of blood, so that the heart fails to obtain sufficient supply of nutrition, which eventually leads to HF [37].

ko04933 (AGE-RAGE signaling pathway in diabetic complications) enriched 10 targets, suggesting that the treatment of diseases may be related to this pathway. Diabetic patients have a higher risk of cardiovascular diseases (such as HF) than healthy adults [38]. The cause of chronic heart failure may be left ventricular stiffness caused by ventricular remodeling. The AGE-RAGE signaling pathway can activate myocardial fibroblasts, resulting in myocardial fibrosis and ventricular remodeling [39].

hsa04066 (HIF-1 signaling pathway) is a signaling pathway that is only activated under hypoxic conditions in the human body. It is an important regulatory pathway of various biological processes in the human body, and the pathway has been proved to have potential value for the treatment of HF [40]. The expression of HIF and its downstream genes can regulate hypoxic cells under hypoxic conditions by regulating mitochondrial metabolism, regulating cell function, and controlling angiogenesis, so as to achieve the effect of protecting cardiomyocytes [41]. Studies have shown that HIF-1 signaling pathway can induce hypoxia-inducible enhancer RNA1 (HERNA1) under hypoxic pathological conditions, and the postdisease development of HERNA1 can protect the normal growth of the left ventricle in mice, regulate its dysfunction, and inhibit the pathological hypertrophy of cardiomyopathy in mice [42].

Both hsa04915 (estrogen signaling pathway) and hsa04020 (calcium signaling pathway) enriched five targets. Estrogen signal is very important in the normal function of the heart. Estrogen signal mainly induces the transcription function of myocardial nucleus in the heart, and estrogen receptor plays an important role in fibroblasts, pulmonary septum and platelets [43]. Obstruction of estrogen circulation increases the risk of neurological and cardiovascular diseases, and estrogen receptors can mediate the cardioprotective function of estrogen by regulating the transcription of related genes or regulating MAPK pathways [31]. Calcium signaling pathway also plays an important role in the treatment of HF. SOCE is an important mechanism in cardiopathy, and aldosterone can promote SOCE in myocardial cells through glucocorticoid receptor and glucocorticoid-regulated kinase 1. At the same time, TRPC channel is also one of the channels that mediate SOCE [32]. Although the current research on the activation of TPRC is not sufficient, it is generally believed that TRPC is related to the Ca^2+^ heart signal and heart disease. The correlation between TPRC pathway and Ca^2+^ signaling pathway in cardiomyocytes has been confirmed to affect the development of cardiac diseases [44]. Studies have shown that the enhancement of SOCE process is related to the increase of Orai 1 expression and the enhancement of cell collagen secretion. Extracellular matrix collagen deposition is the main inducing factor for the development of HF [45].

In this study, the key active ingredients and core targets were docked at molecular level, and the results showed that the key active ingredients, such as quercetin and isorhamnetin, were able to successfully dock with ADRB2 and HMOX1 with a total score above 5.0, suggesting that these key components have strong binding force with the targets. This work provides important basis for exploring the integrative mechanism of Tinglizi against chronic heart failure from multiple components and multiple targets.

With the help of network pharmacology and molecular docking technology, this study speculates the potential role of Tinglizi in the synergistic treatment of CHF through multicomponent, multitarget, and multichannel, which reflects the integrity of TCM. However, there are some limitations in this study. For example, this study relies only on data analysis. Relevant experiments can be carried out in the future to further verify this study.

## 5. Conclusion

Through network pharmacology and molecular docking technology, we found that the main components of Tinglizi in the treatment of CHF are quercetin, kaempferol, *β*-Sitosterol, isorhamnetin, and so on. The action targets are ADRB2, HMOX1, SLC6A2, NOS2, DPP4, NOS3, RELA, XDH, OPRM1, BCL2, BAX, TGFB1, PON1, NR3C2, MAPK8, and so on. The main pathways are AGE-RAGE signaling pathway in diabetic complications, HIF-1 signaling pathway, estrogen signaling pathway, and so on. They play an integrated role in the treatment of CHF.

## Figures and Tables

**Table 1:** Effective components of Tinglizi

Mol ID	Molecule name	OB (%)	DL
MOL002211	11,14-Eicosadienoic acid	39.99	0.2
MOL000296	Hederagenin	36.91	0.75
MOL000354	Isorhamnetin	49.6	0.31
MOL000358	*β*-Sitosterol	36.91	0.75
MOL003905	K-STROPHANTHOSIDE	70.65	0.22
MOL003906	K-STROPHANTHOSIDE_qt	30.8	0.78
MOL003907	Erysimoside	65.45	0.23
MOL003908	Cynotoxin	99.94	0.78
MOL003909	Evobioside	44.25	0.24
MOL003927	Dihomolinolenic acid	44.11	0.2
MOL000422	Kaempferol	41.88	0.24
MOL000098	Quercetin	46.43	0.28

**Table 2:** Top ten action targets of degree.

Target gene	Target protein	Degree
IL-6	Interleukin-6	15
TP53	Tumor protein 53	14
MMP9	Matrix metalloproteinase 9	13
MAPK8	Mitogen-activated protein kinase 8	12
VEGFA	Vascular endothelial growth factor A	12
CCL2	Chemokine 2	10
HMOX1	Heme oxygenase 1	9
MMP2	Matrix metalloproteinase 2	9
RELA	Nuclear factor *κ*Bp65	9
ERBB2	Receptor protein tyrosine kinase 2	7

**Table 3:** KEGG pathway enrichment results.

Pathway	Name	Number of targets	p.adjust
hsa05418	Fluid shear stress and atherosclerosis	12	1.00*E* − 12
hsa04933	AGE-RAGE signaling pathway in diabetic complications	10	3.27*E* − 11
hsa04066	HIF-1 signaling pathway	9	2.35*E* − 09
hsa05417	Lipid and atherosclerosis	10	3.56*E* − 08
hsa05212	Pancreatic cancer	7	1.31*E* − 07
hsa04926	Relaxin signaling pathway	8	1.70*E* − 07
hsa01522	Endocrine resistance	7	5.65*E* − 07
hsa05142	Chagas disease	7	6.55*E* − 07
hsa05161	Hepatitis B	8	6.83*E* − 07
hsa05152	Tuberculosis	8	1.40*E* − 06
hsa05219	Bladder cancer	5	3.11*E* − 06
hsa04657	IL-17 signaling pathway	6	7.49*E* − 06
hsa05146	Amoebiasis	6	1.12*E* − 05
hsa05145	Toxoplasmosis	6	1.81*E* − 05
hsa04071	Sphingolipid signaling pathway	6	2.42*E* − 05
hsa05415	Diabetic cardiomyopathy	7	3.61*E* − 05
hsa05162	Measles	6	5.23*E* − 05
hsa01521	EGFR tyrosine kinase inhibitor resistance	5	5.23*E* − 05
hsa05210	Colorectal cancer	5	7.54*E* − 05
hsa04932	Nonalcoholic fatty liver disease	6	8.41*E* − 05
hsa05222	Small cell lung cancer	5	9.51*E* − 05
hsa05323	Rheumatoid arthritis	5	9.57*E* − 05
hsa05215	Prostate cancer	5	0.000112447
hsa05144	Malaria	4	0.000150485
hsa04659	Th17 cell differentiation	5	0.000167799
hsa04931	Insulin resistance	5	0.000167799
hsa04668	TNF signaling pathway	5	0.000192705
hsa05167	Kaposi sarcoma-associated herpesvirus infection	6	0.000215762
hsa04010	MAPK signaling pathway	7	0.0002249
hsa04722	Neurotrophin signaling pathway	5	0.000232382
hsa05169	Epstein-Barr virus infection	6	0.000244682
hsa05205	Proteoglycans in cancer	6	0.000257499
hsa05321	Inflammatory bowel disease	4	0.000310948
hsa05166	Human T-cell leukemia virus 1 infection	6	0.000377637
hsa04210	Apoptosis	5	0.000377637
hsa05163	Human cytomegalovirus infection	6	0.000382944
hsa04915	Estrogen signaling pathway	5	0.000382944
hsa01524	Platinum drug resistance	4	0.000426283
hsa05133	Pertussis	4	0.000474104
hsa05166	Human T-cell leukemia virus 1 infection	6	0.000377637

## Data Availability

The data used to support the findings of this study are available from the corresponding author upon request.

## Supplementary Materials

Supplementary Materials Supplementary Table 1: the binding site between the compounds and the targets.
