# Supplementary material for: Pharmacological Mechanisms of Tinglizi against Chronic Heart Failure Determined by Network Pharmacology and Molecular Docking
Source: Evid Based Complement Alternat Med. 2022 Jan 6;2022:2152399. doi: 10.1155/2022/2152399 (PMC8758258; doi:10.1155/2022/2152399)
Supplement: Supplementary Materials — Supplementary Table 1: the binding site between the compounds and the targets. [file 2152399.f1.docx]

**Supplementary table 1. The binding site between the compounds and the targets**

| **Compounds** | **Target** | | **Molecular docking diagram** | | |
| --- | --- | --- | --- | --- | --- |
| Quercetin | MMP9 | | 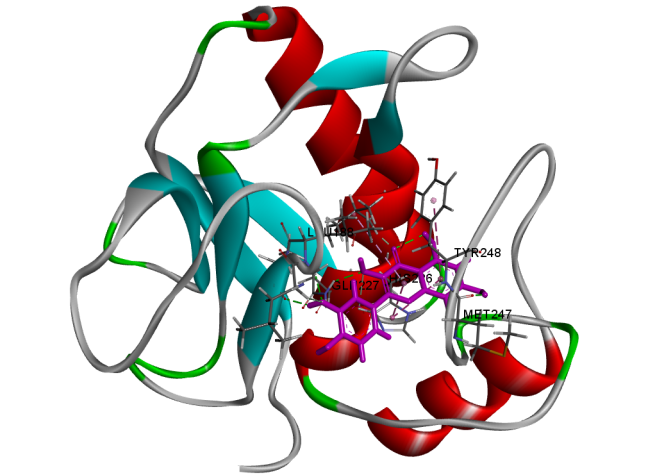 | | 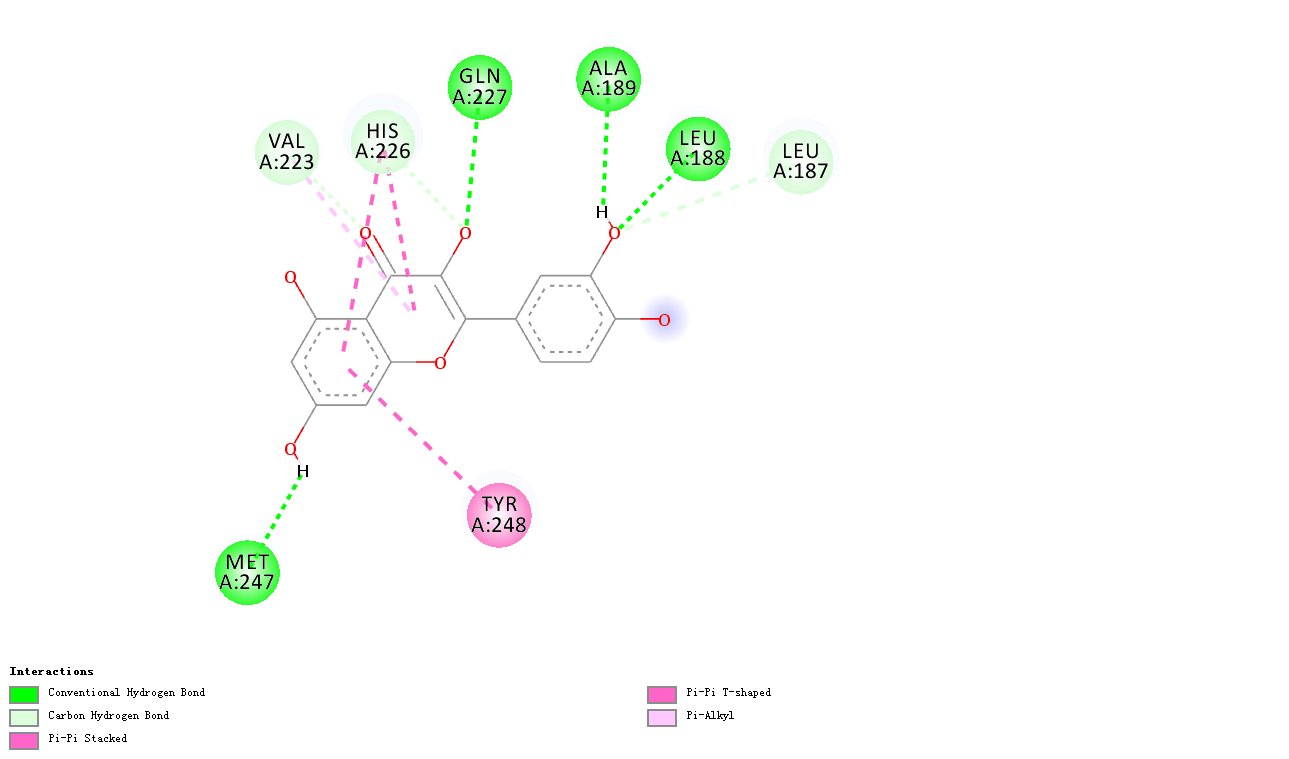 |
|  | PPARA | | 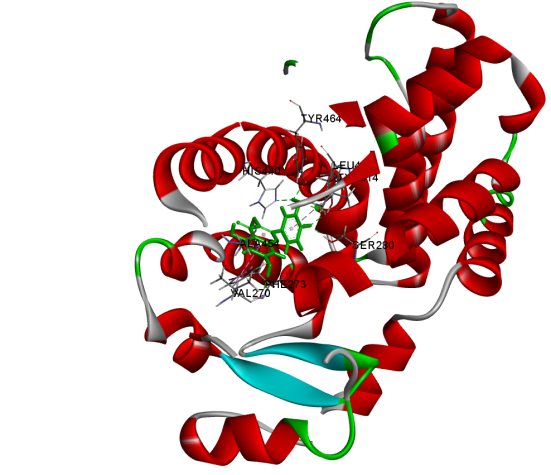 | | 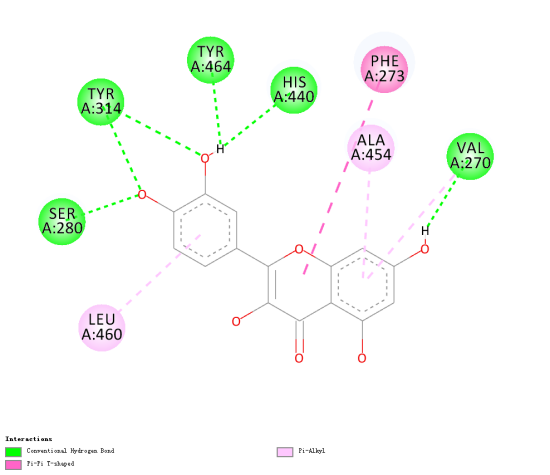 |
|  | HMOX1 | | 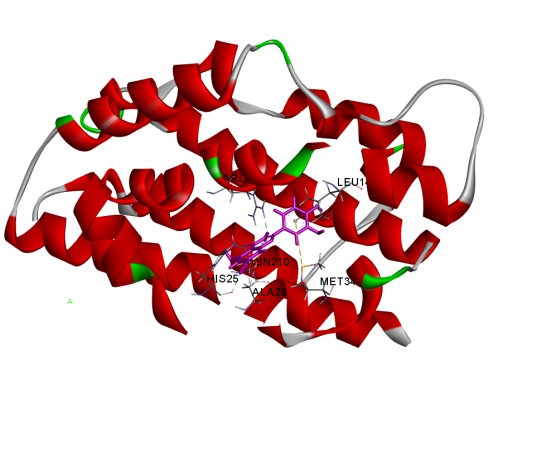 | | 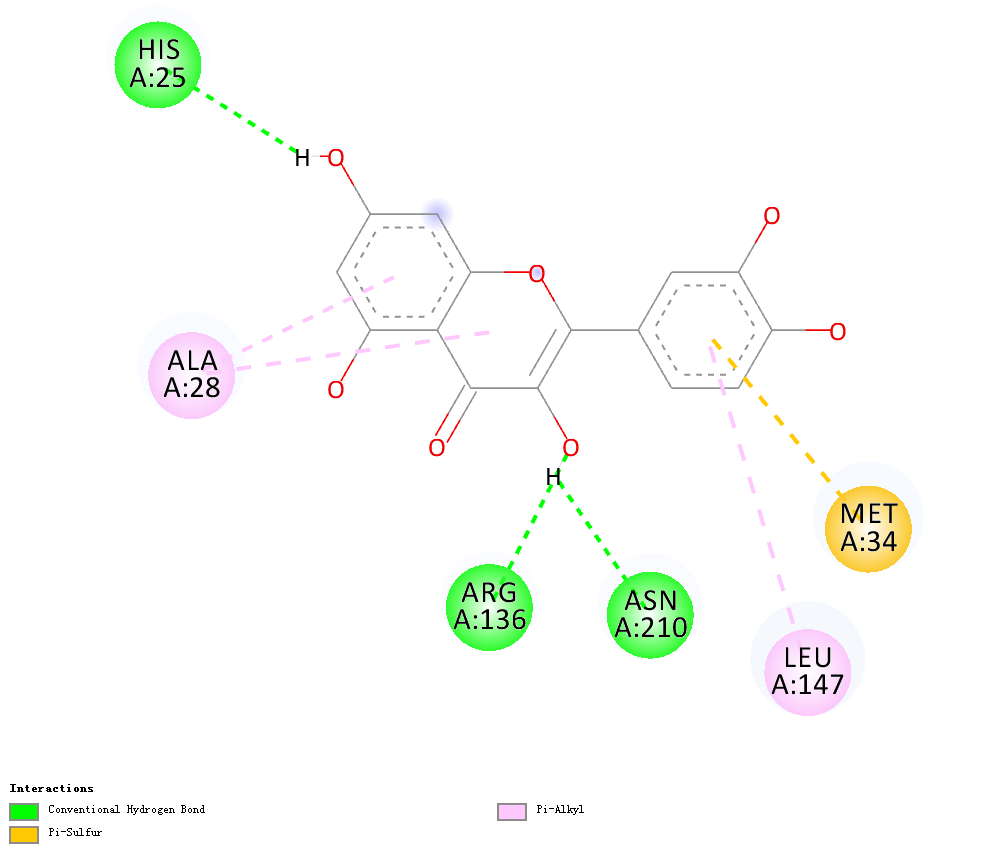 |
|  | OPRM1 | | 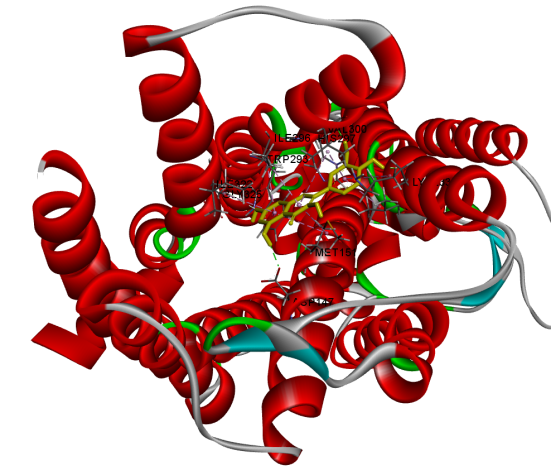 | | 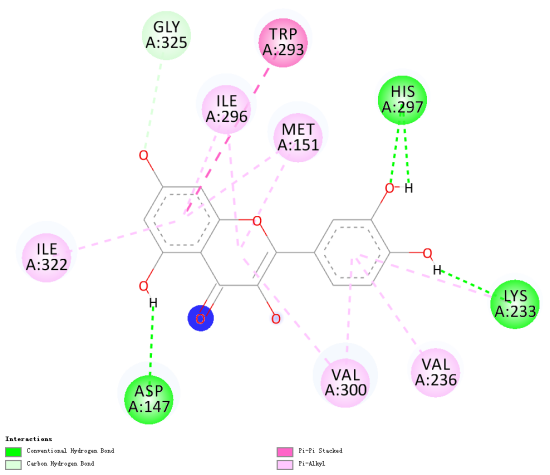 |
|  | NRC32 | | 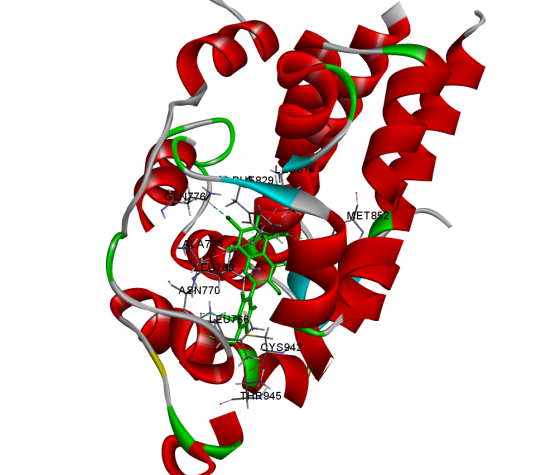 | | 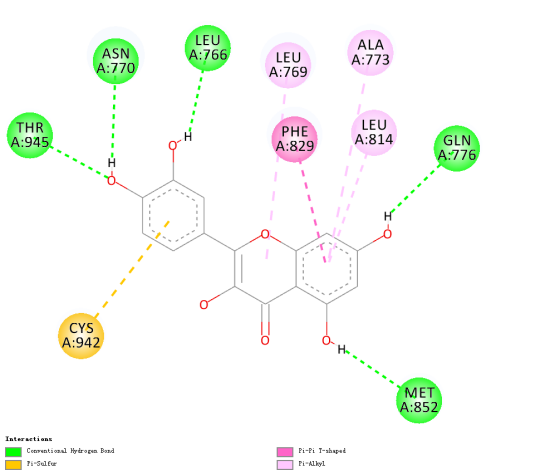 |
|  | ADRB2 | | 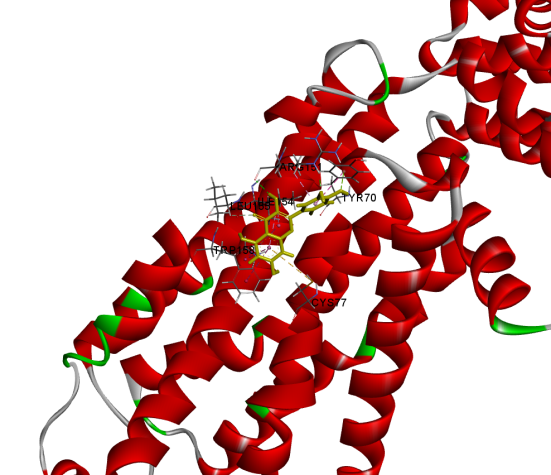 | | 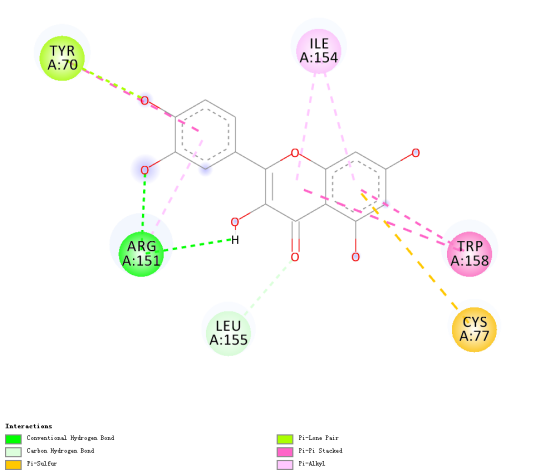 |
|  | XDH | | 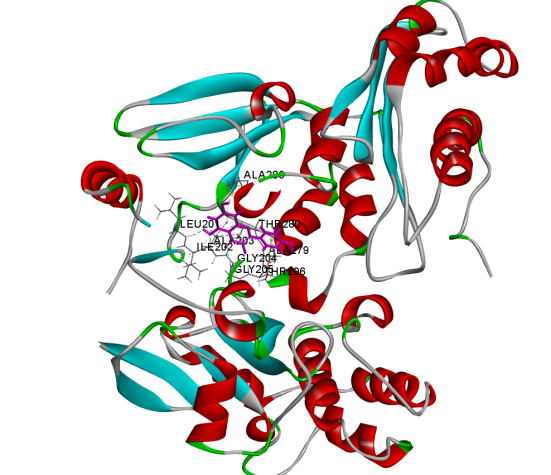 | | 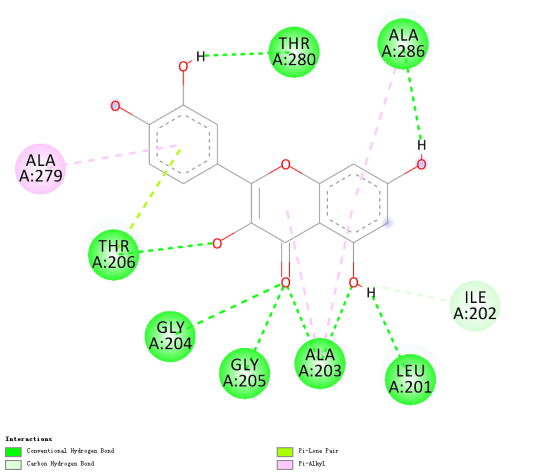 |
|  | DPP4 | | 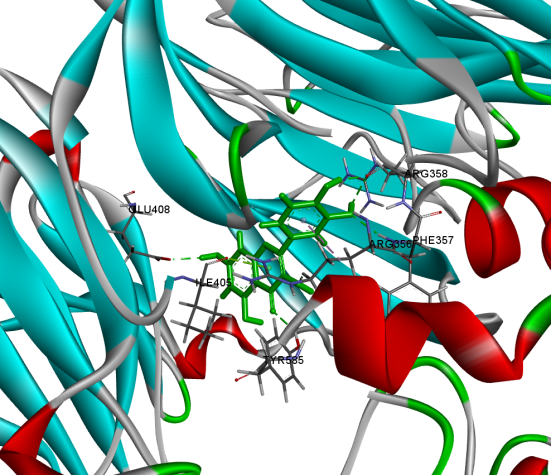 | | 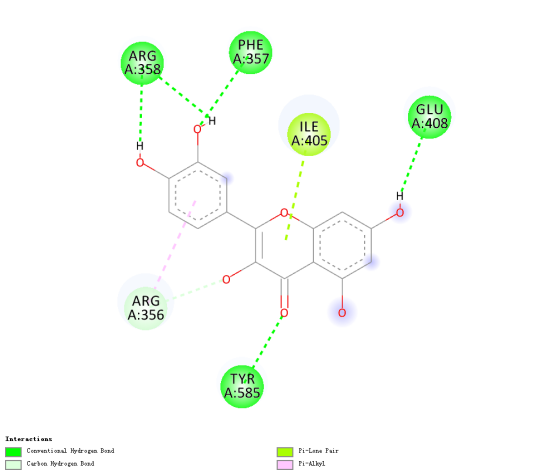 |
| Hederagenin | | MMP9 | | 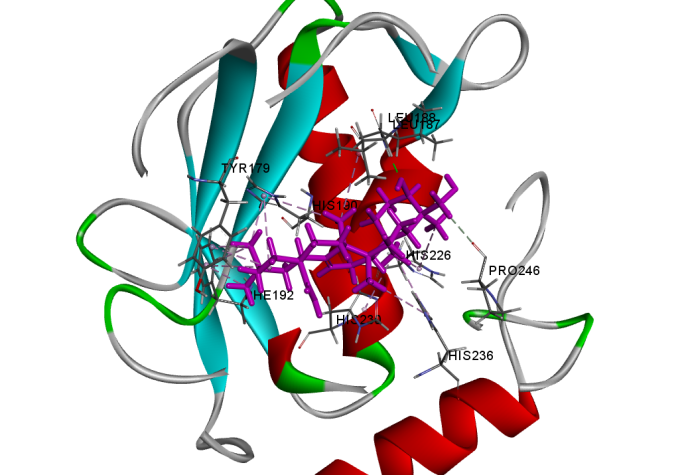 | 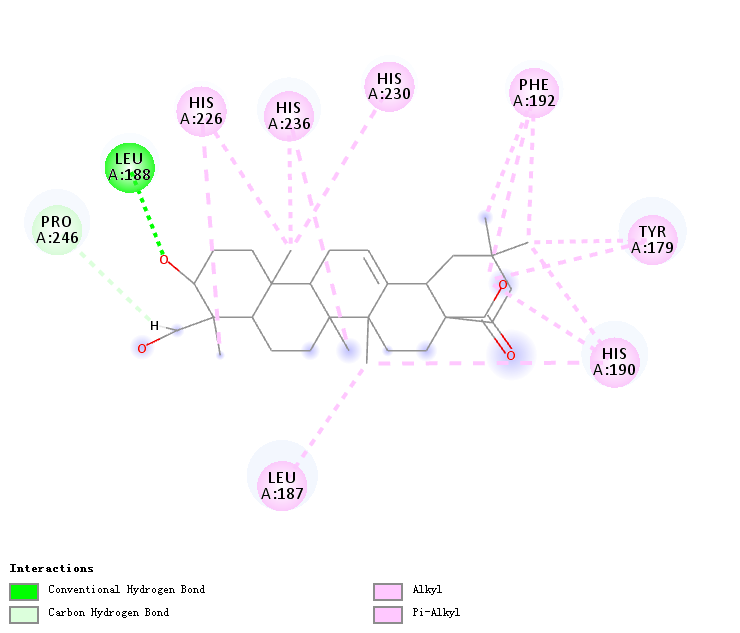 |
|  |  | PPARA | | 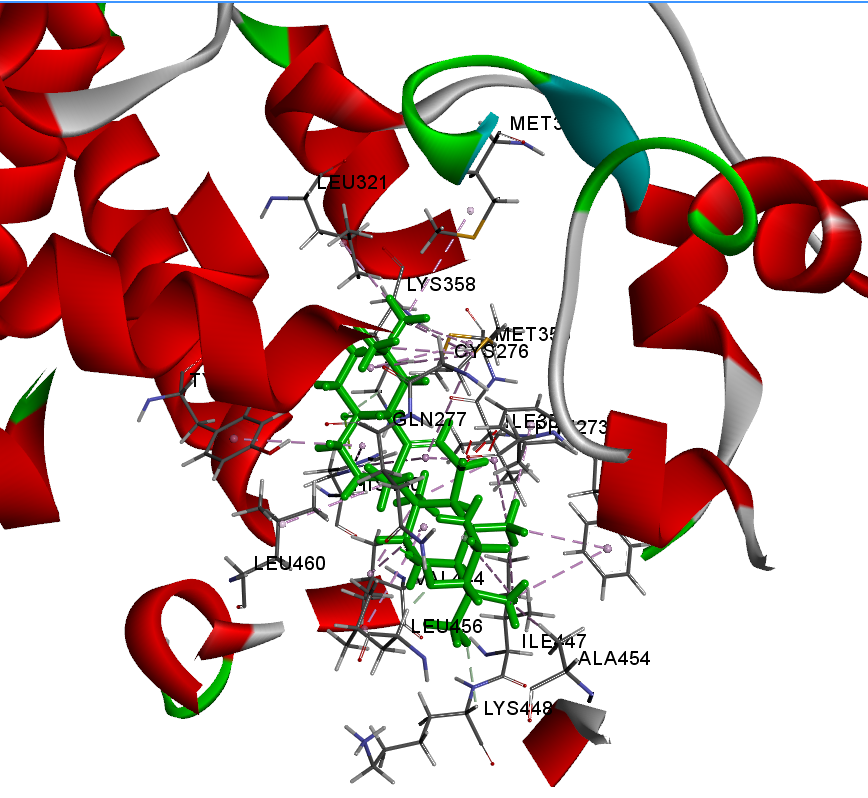 | 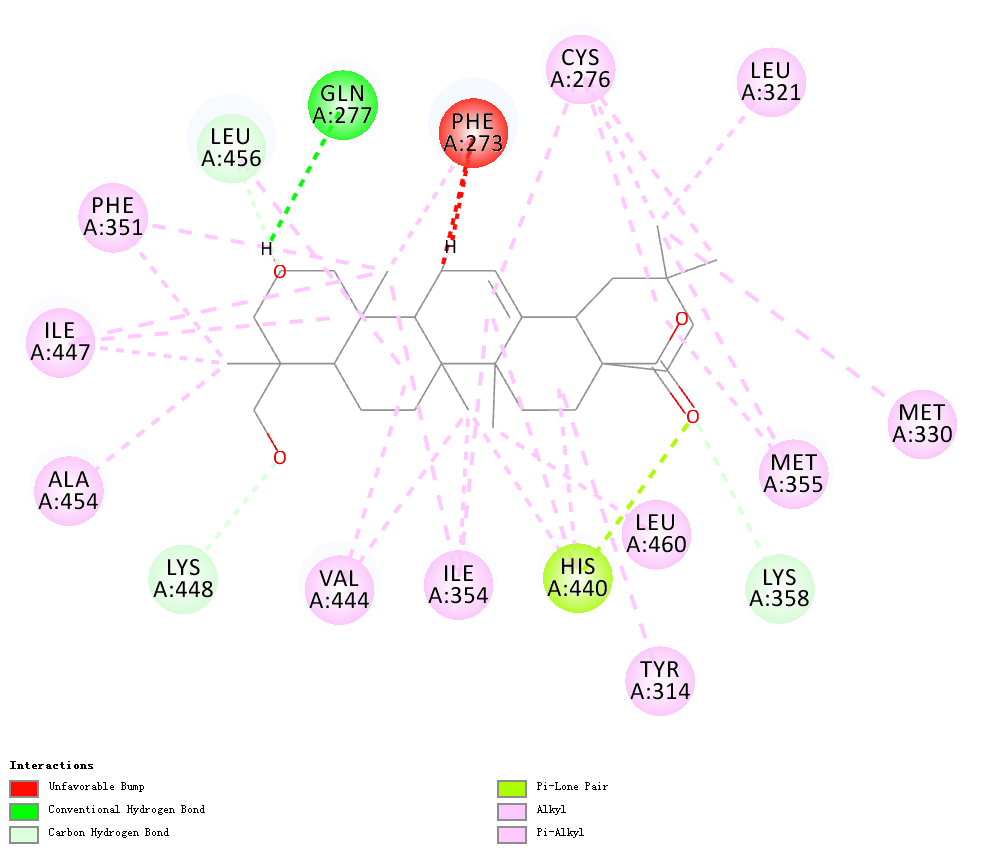 |
|  |  | HMOX1 | | 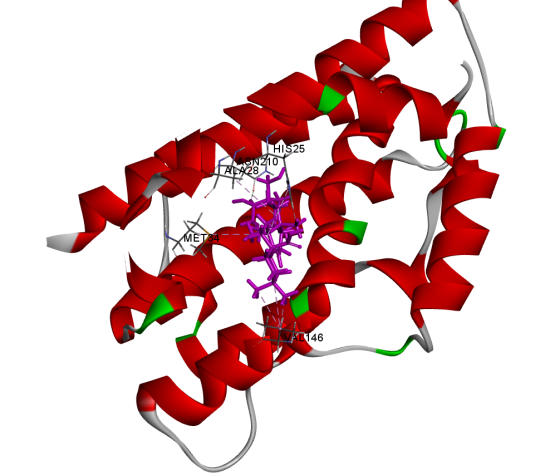 | 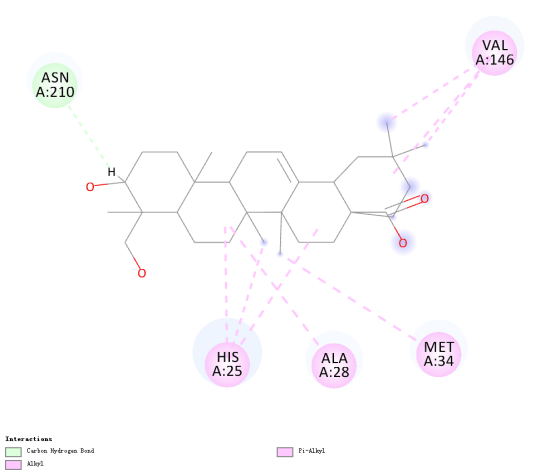 |
|  |  | OPRM1 | | 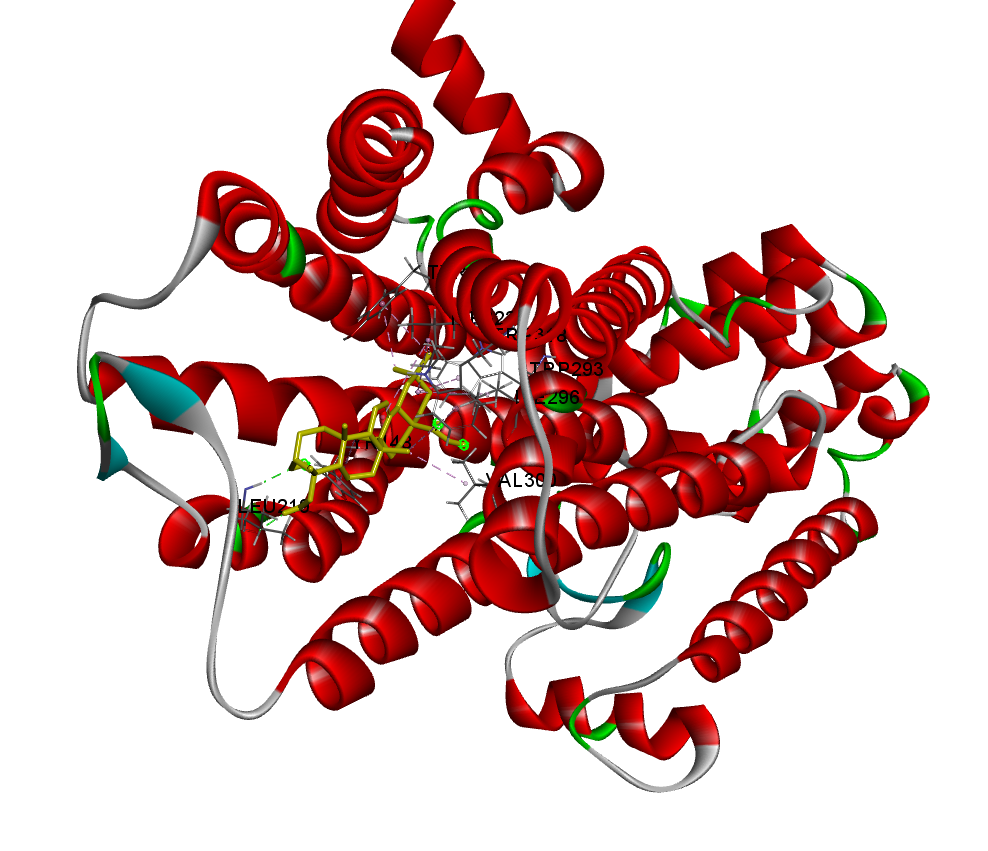 | 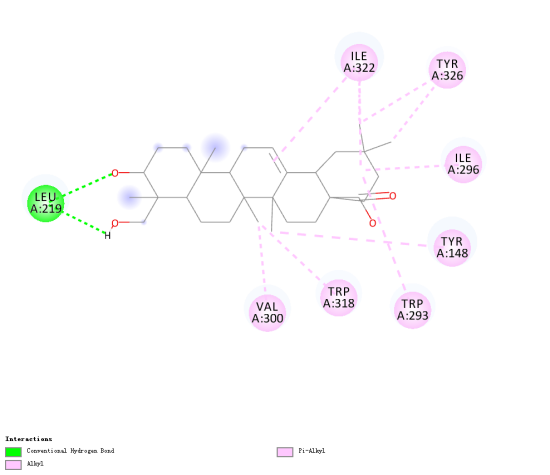 |
|  |  | NRC32 | | 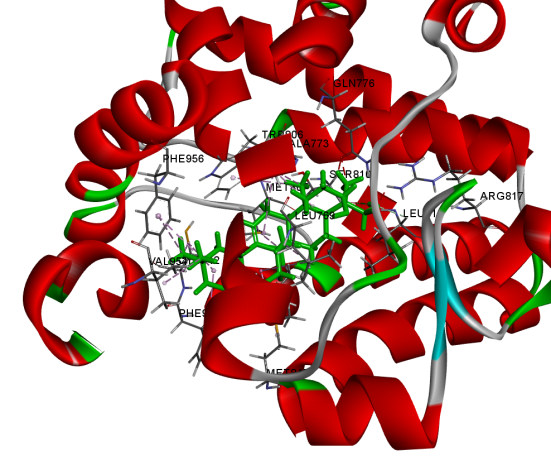 | 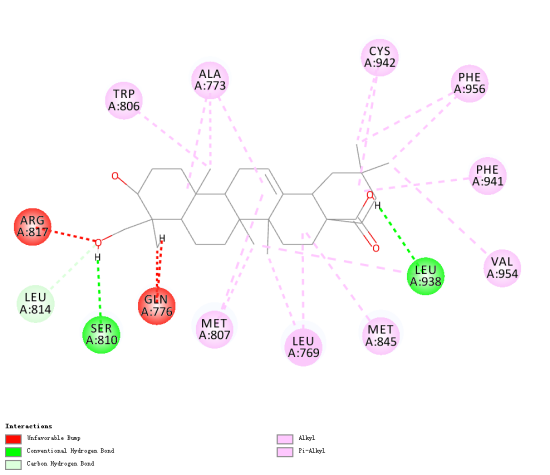 |
|  |  | ADRB2 | | 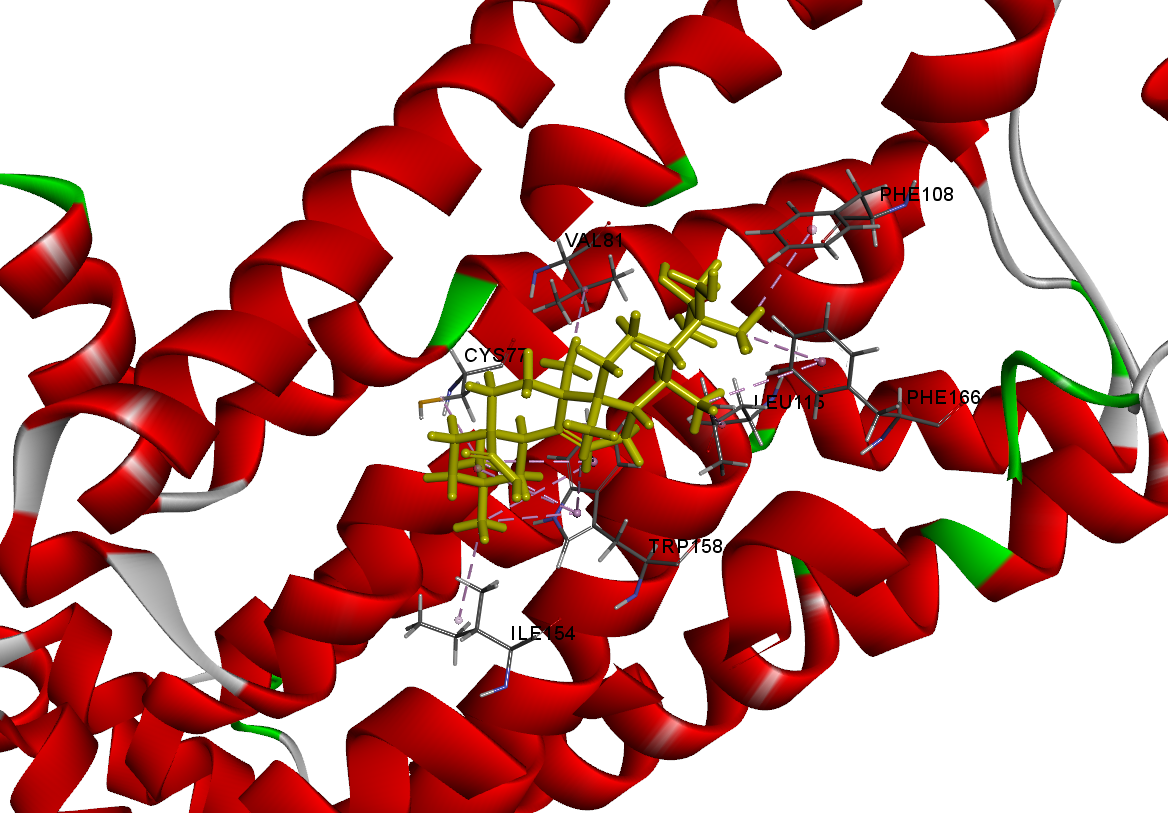 | 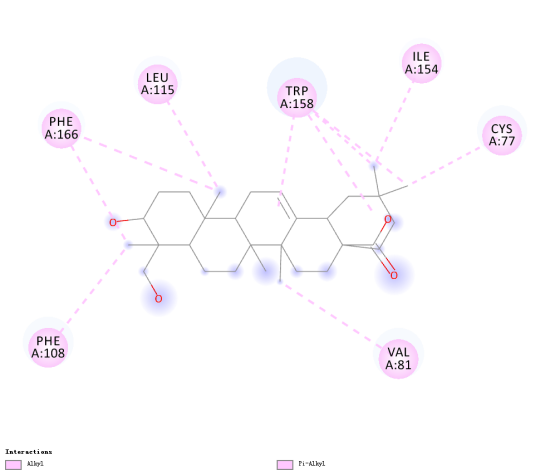 |
|  |  | XDH | | 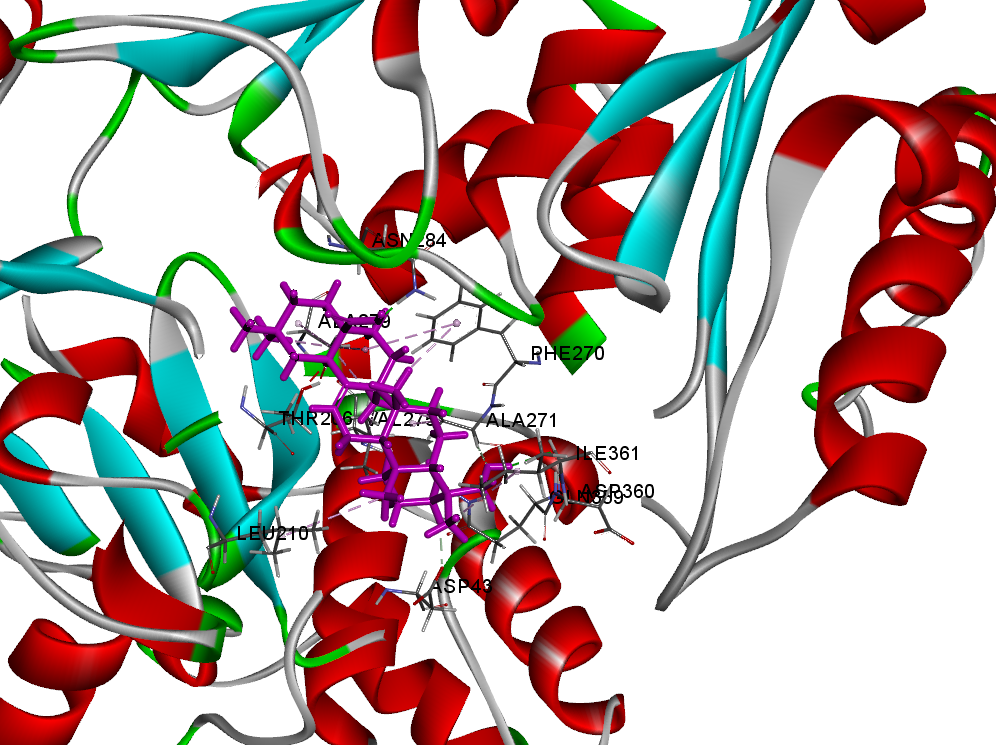 | 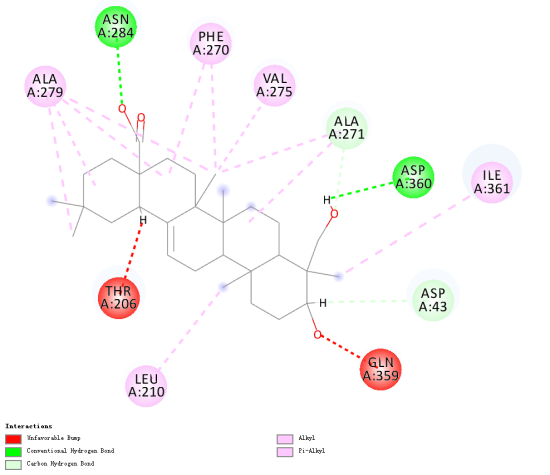 |
|  |  | DPP4 | | 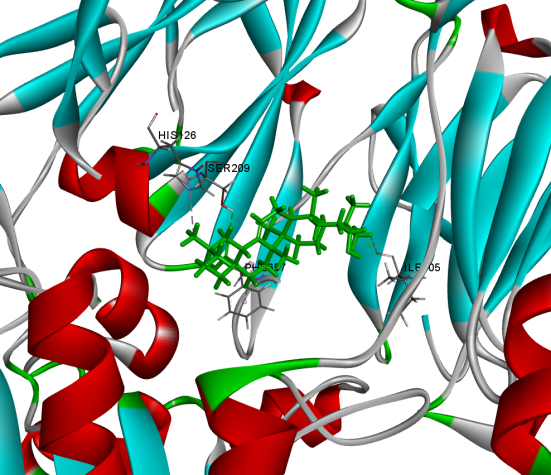 | 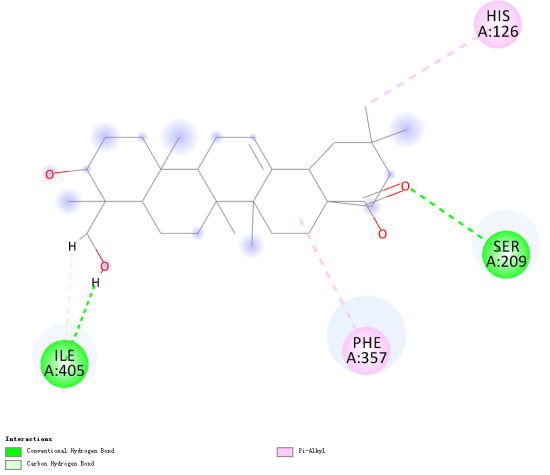 |
| Isorhamnetin | | MMP9 | | 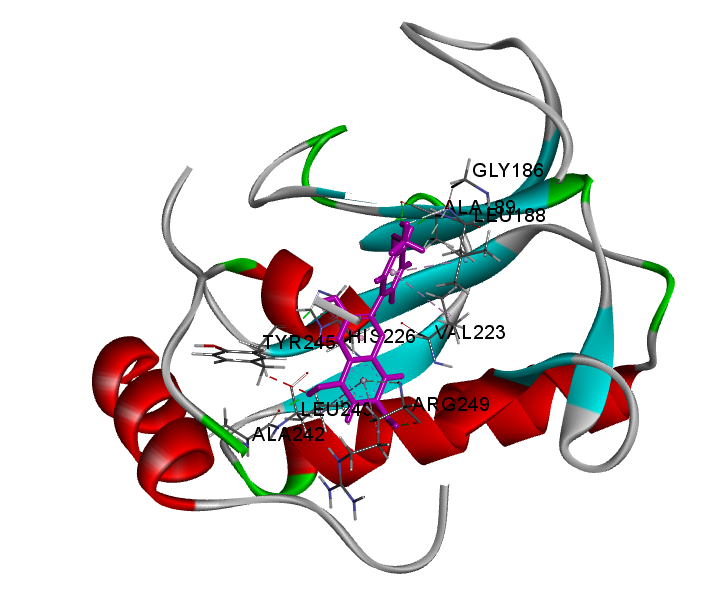 | 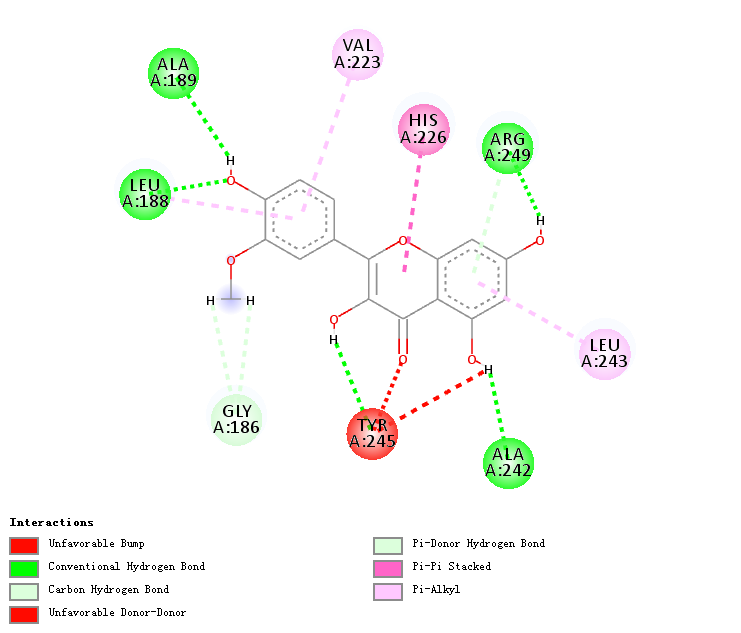 |
|  | | PPARA | | 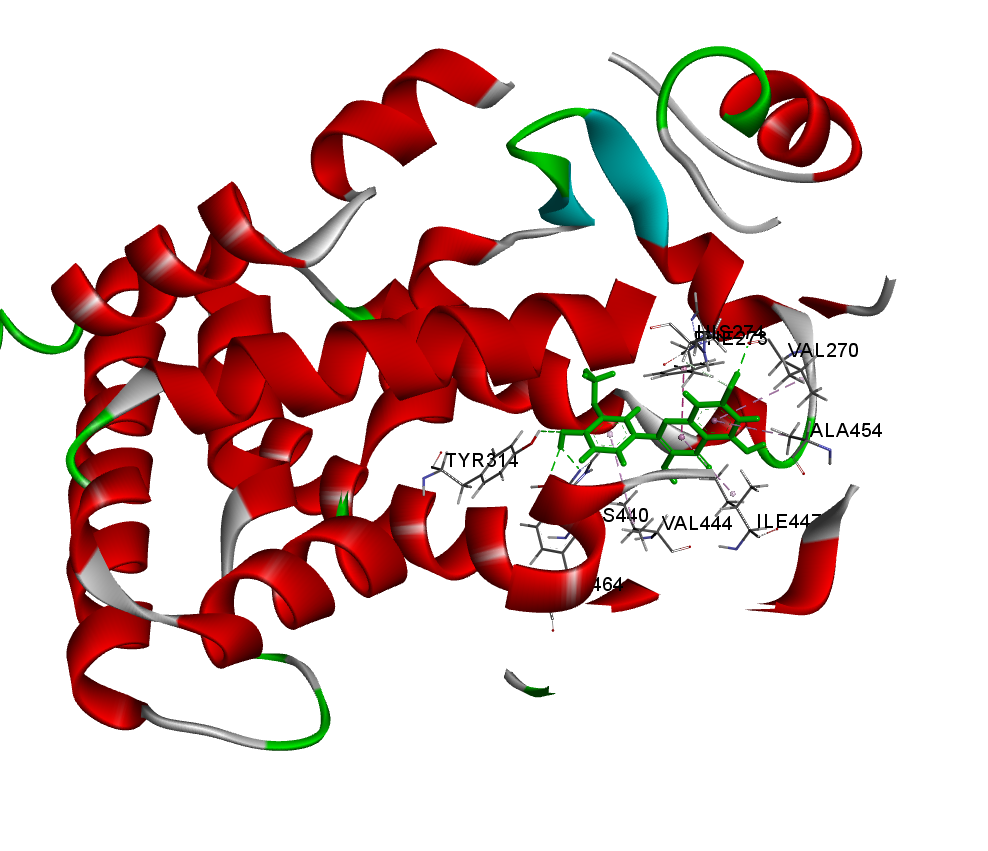 | 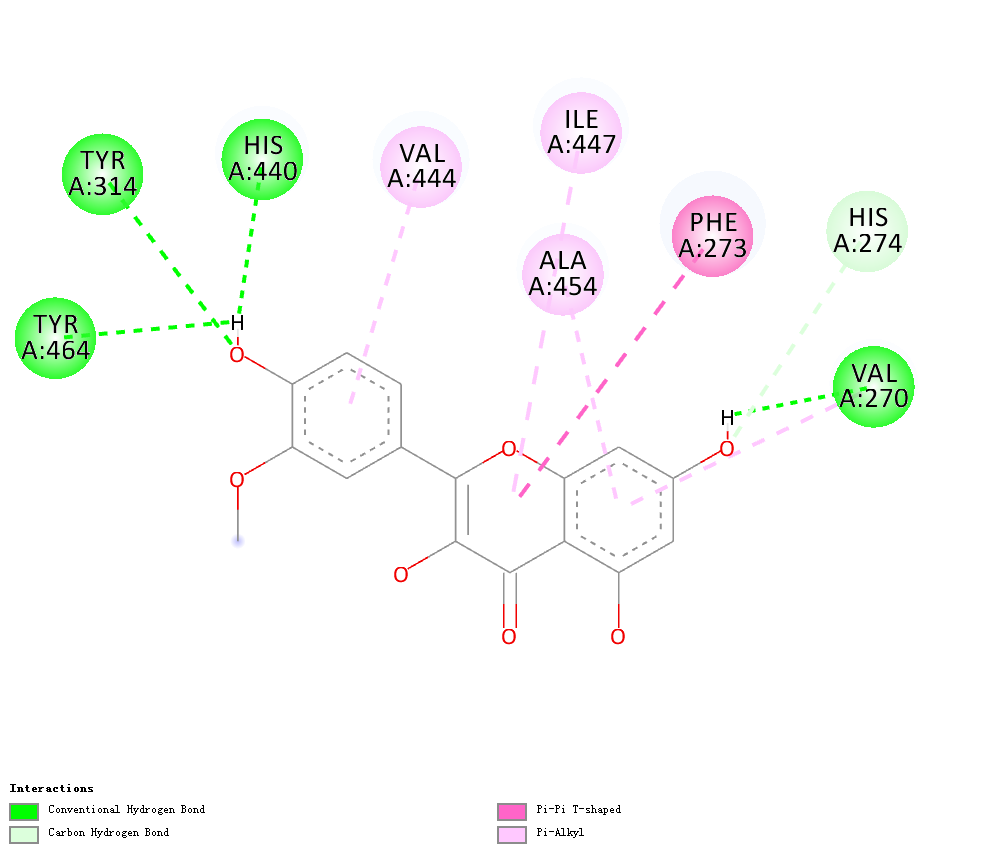 |
|  | | HMOX1 | | 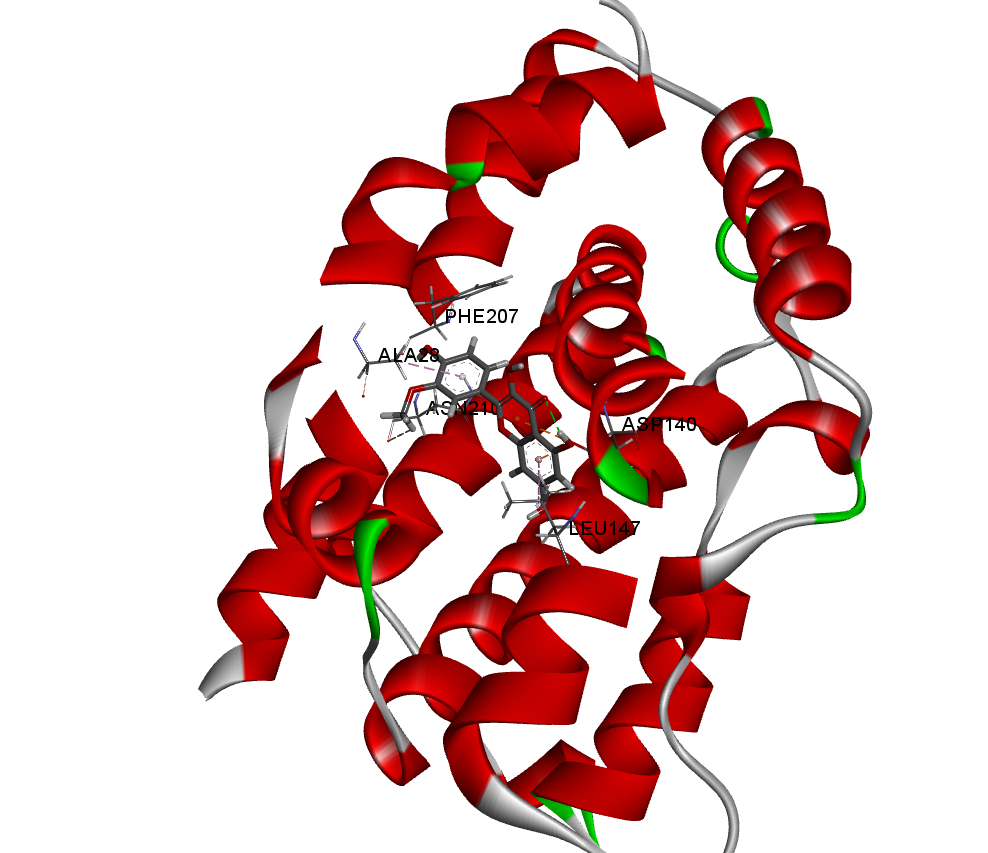 | 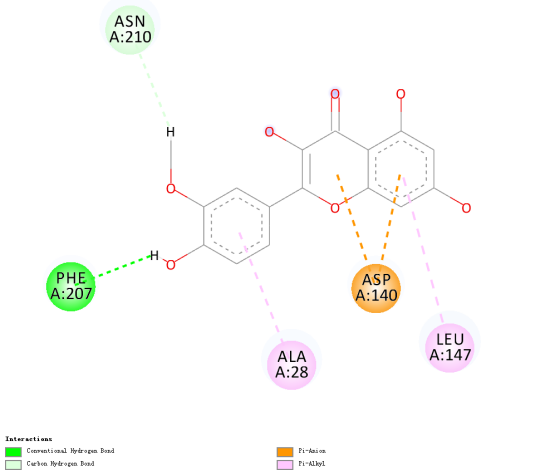 |
|  | | OPRM1 | | 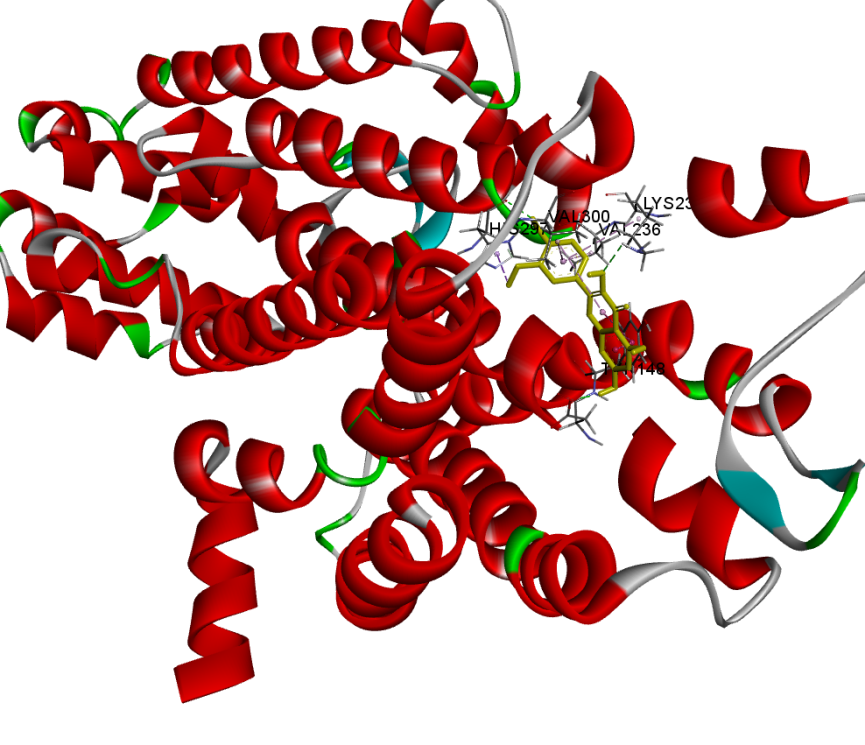 | 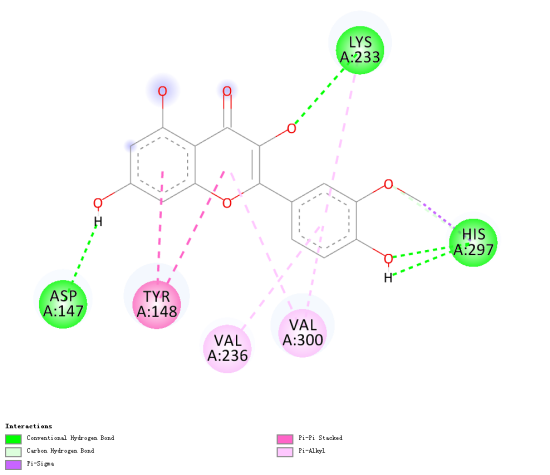 |
|  | | NRC32 | | 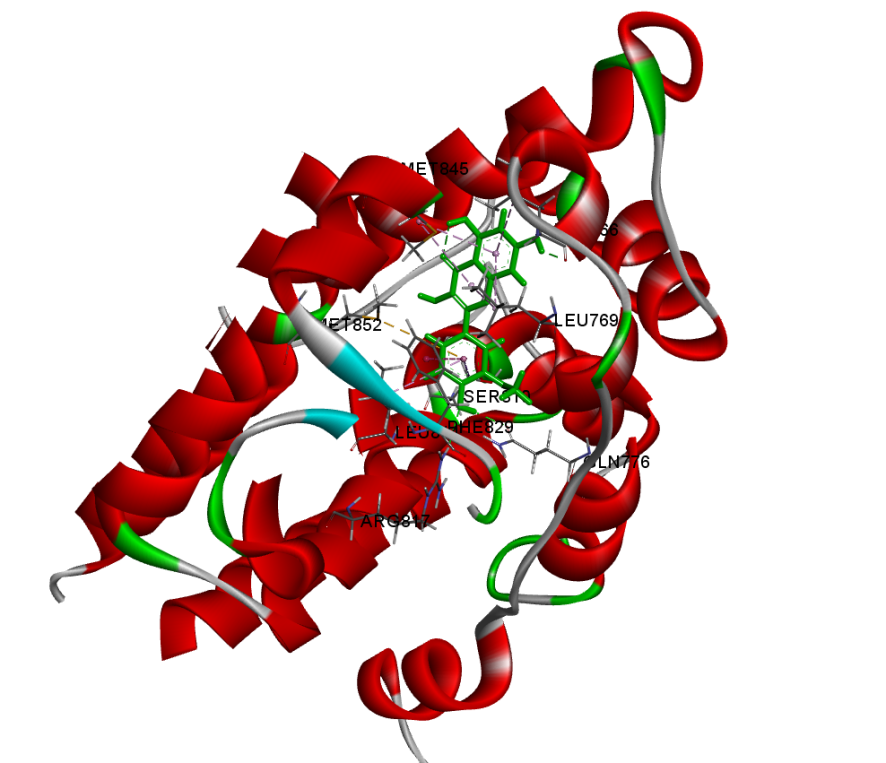 | 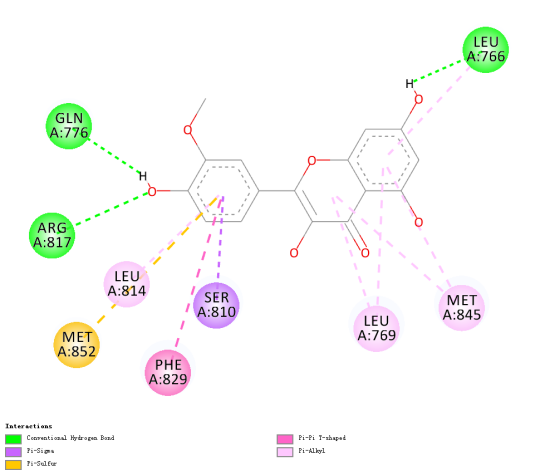 |
|  | | ADRB2 | | 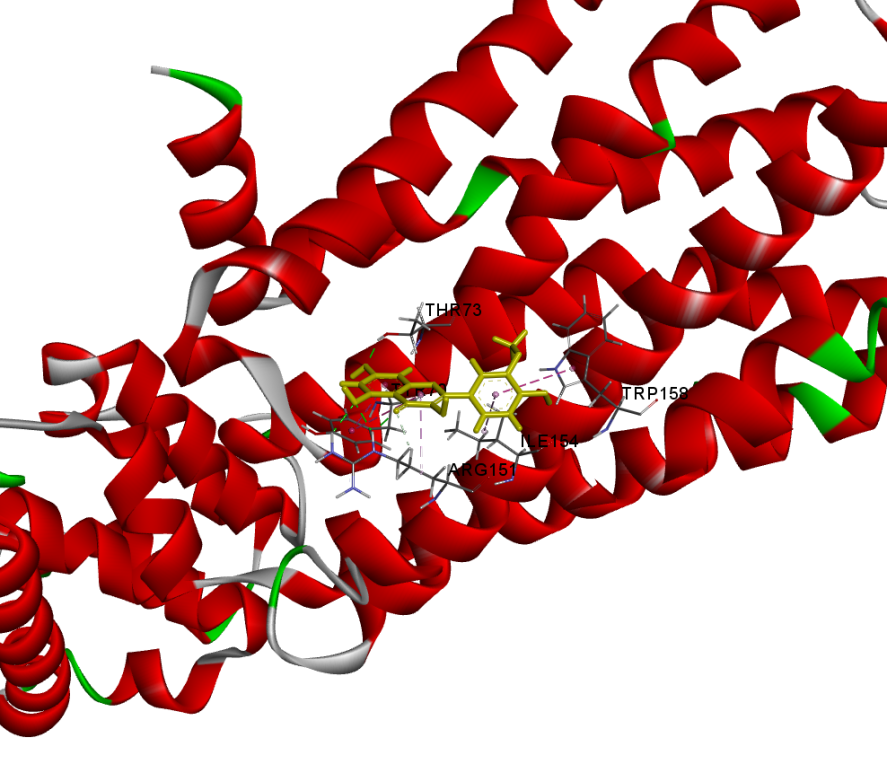 | 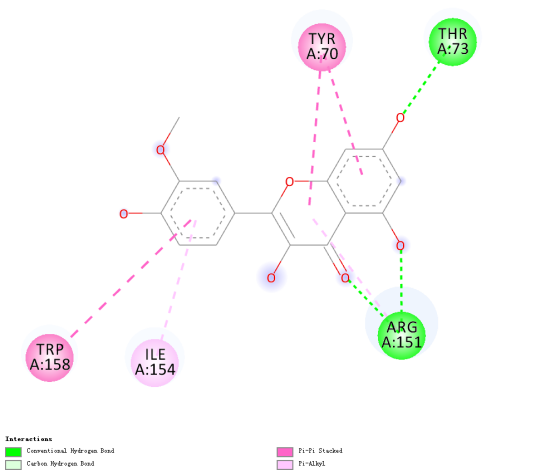 |
|  | | XDH | | 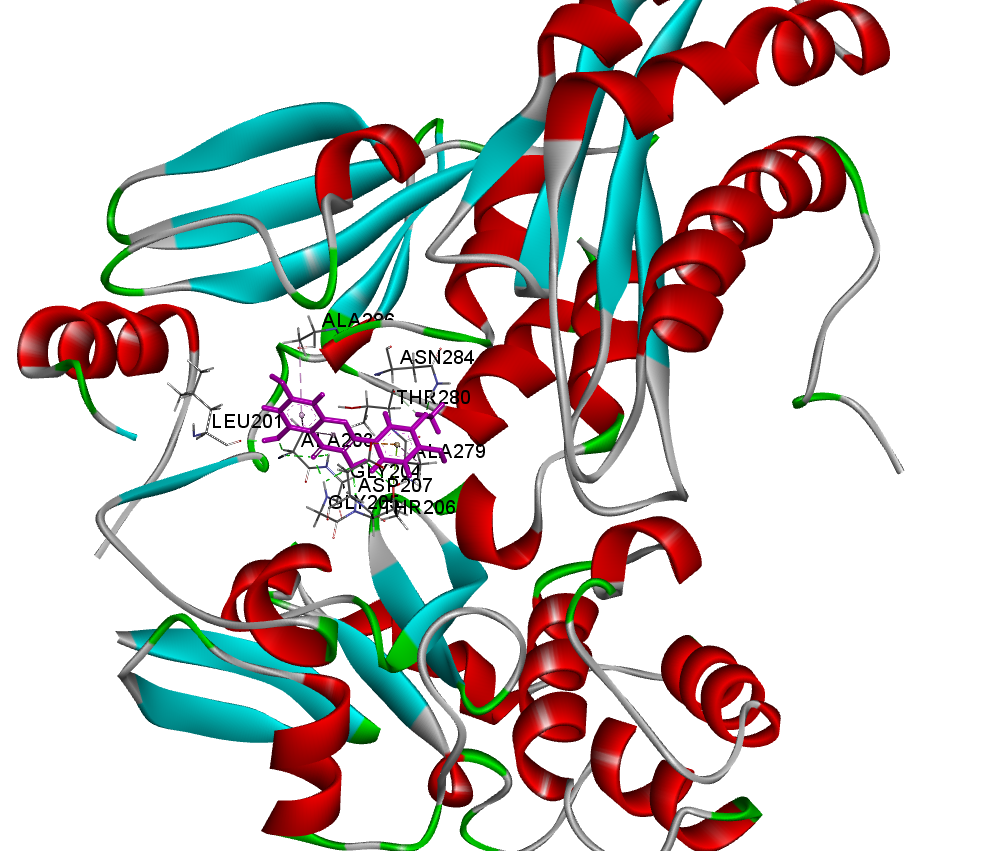 | 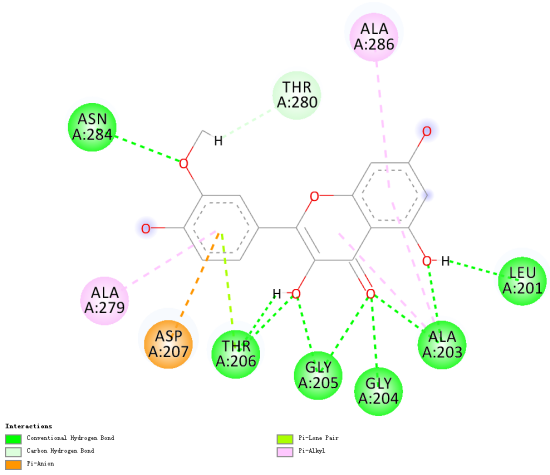 |
|  | | DPP4 | | 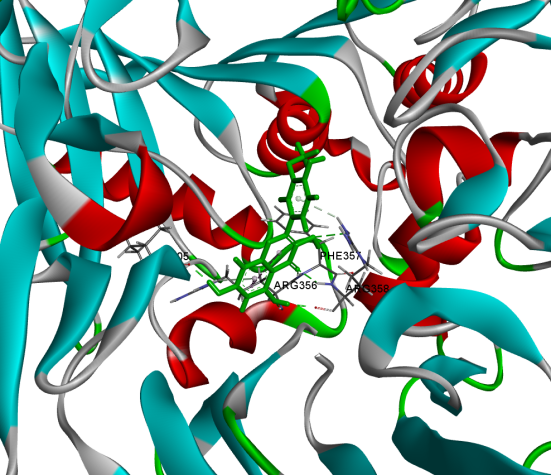 | 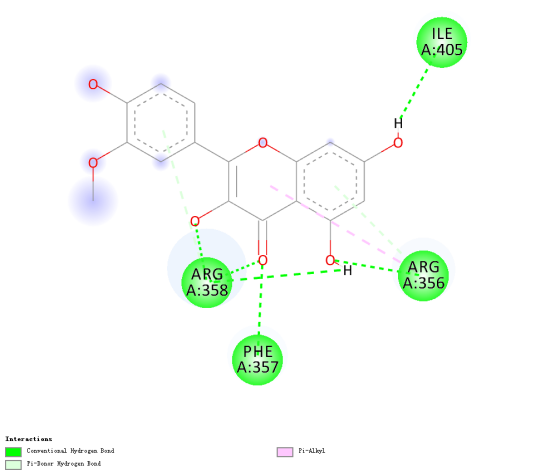 |
| Kaempferol | | MMP9 | | 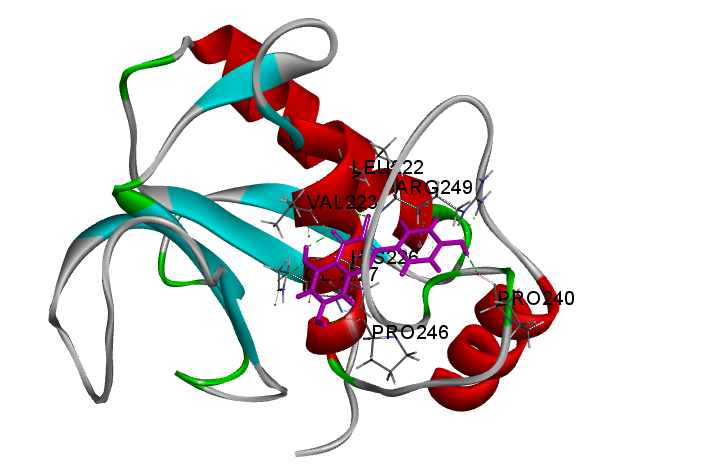 | 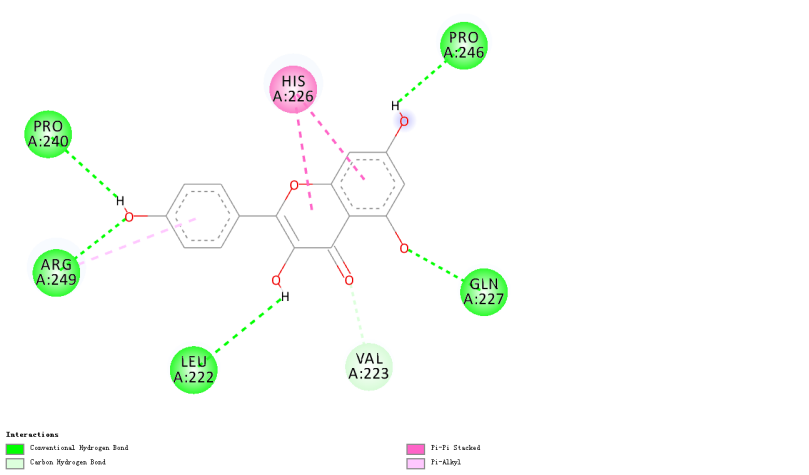 |
|  | | PPARA | | 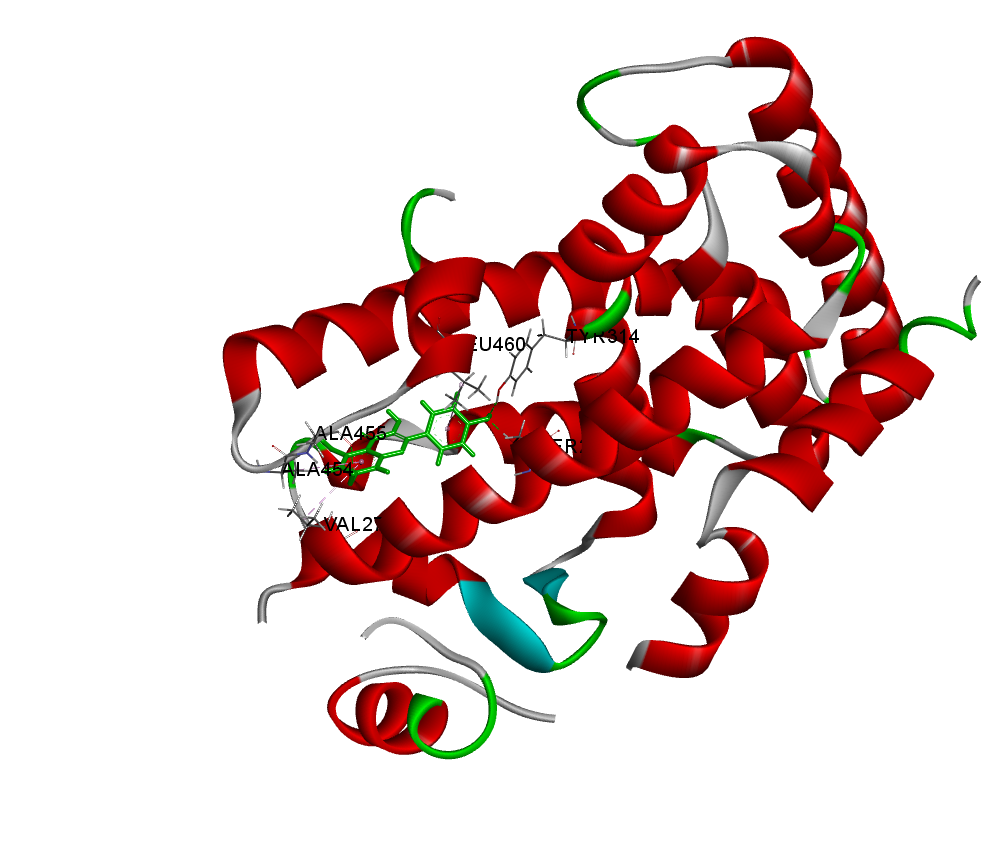 | 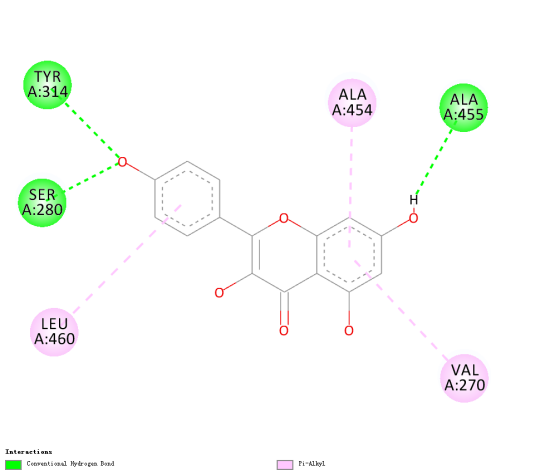 |
|  | | HMOX1 | | 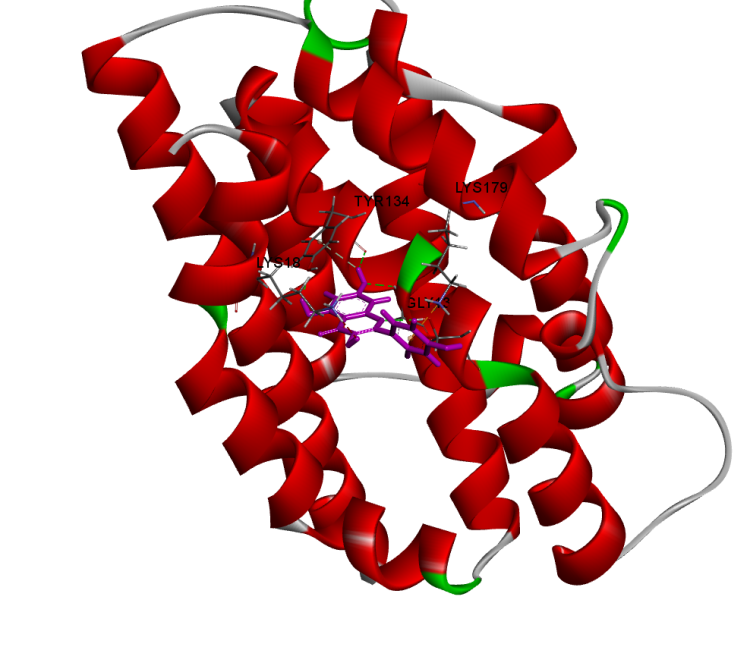 | 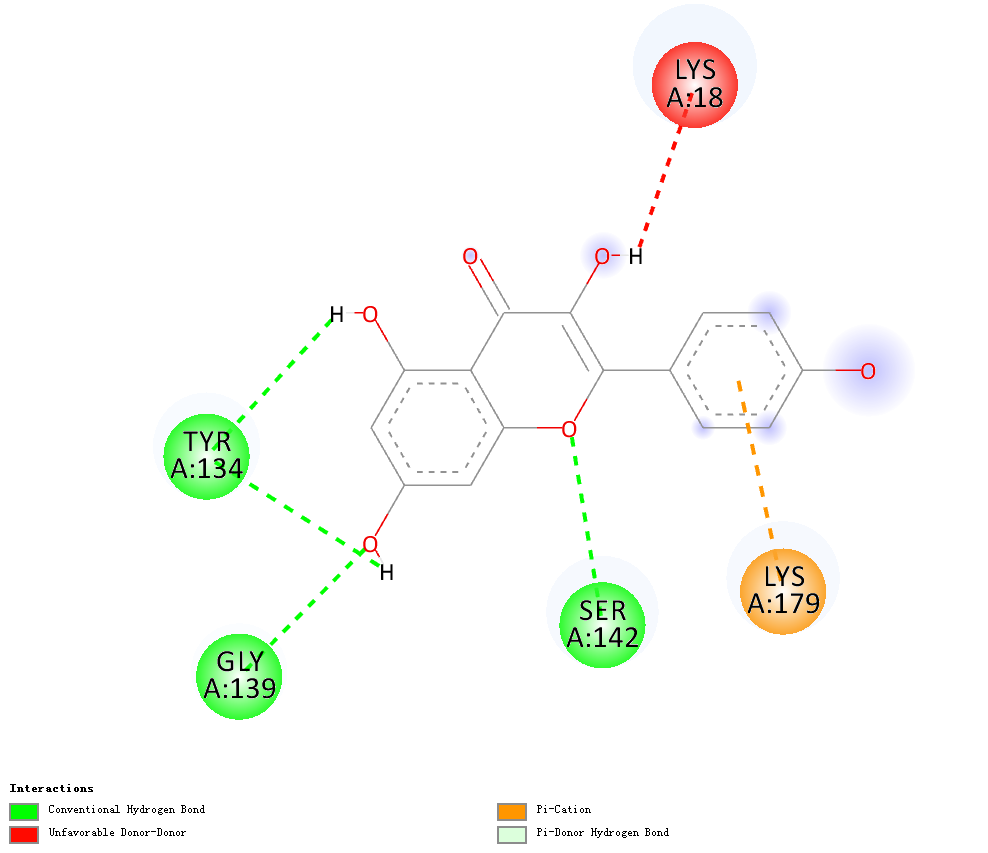 |
|  | | OPRM1 | | 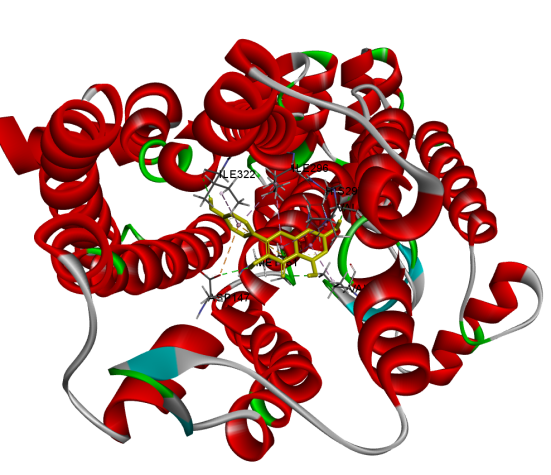 | 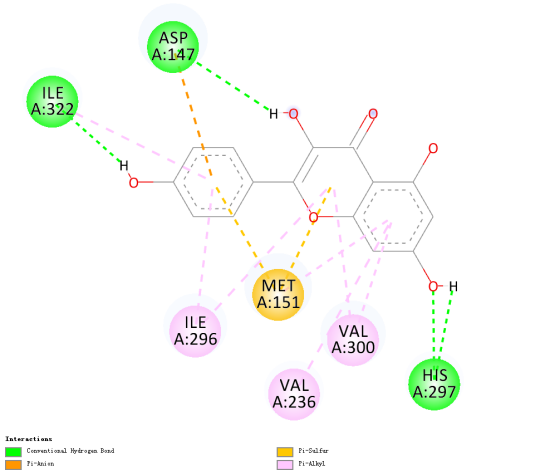 |
|  | | NRC32 | | 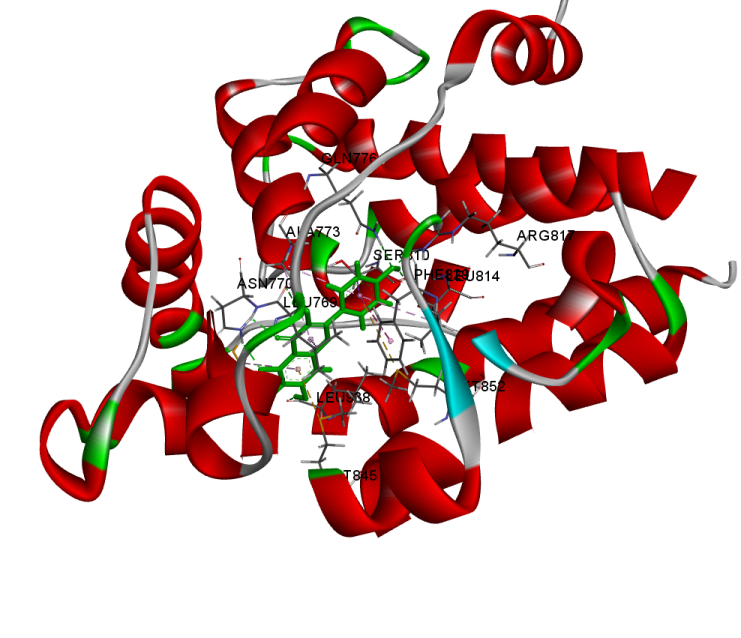 | 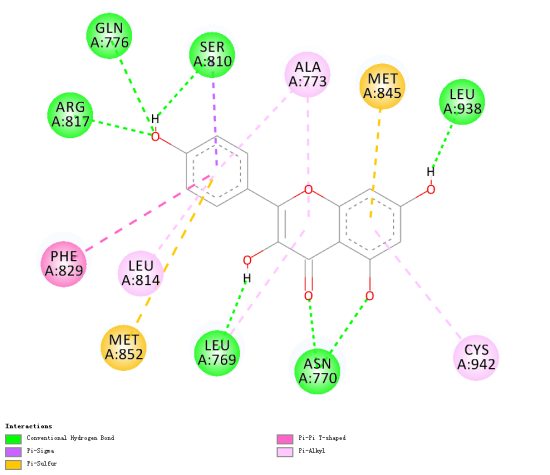 |
|  | | ADRB2 | | 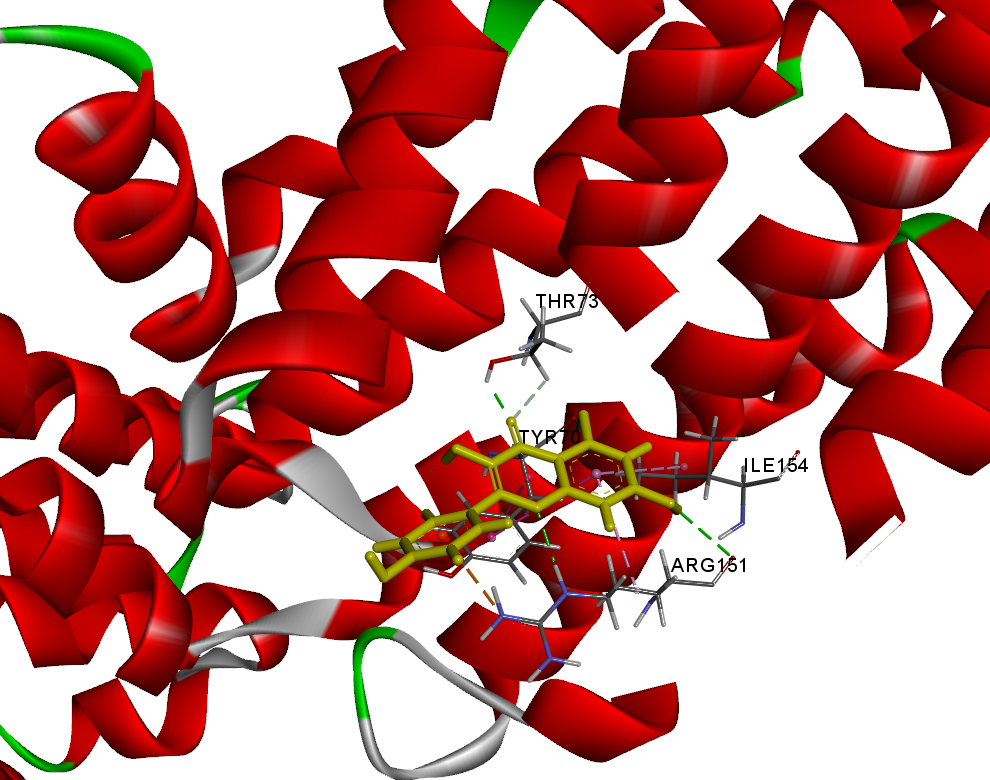 | 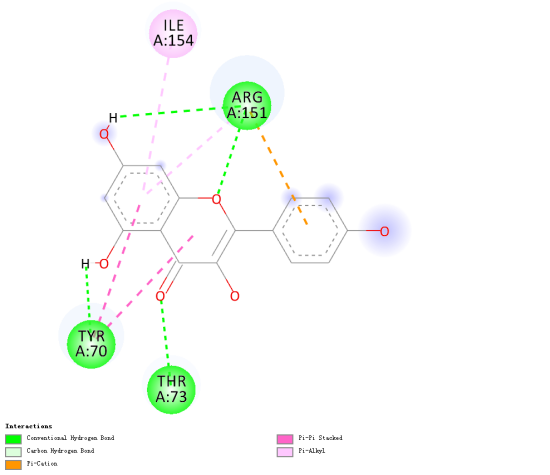 |
|  | | XDH | | 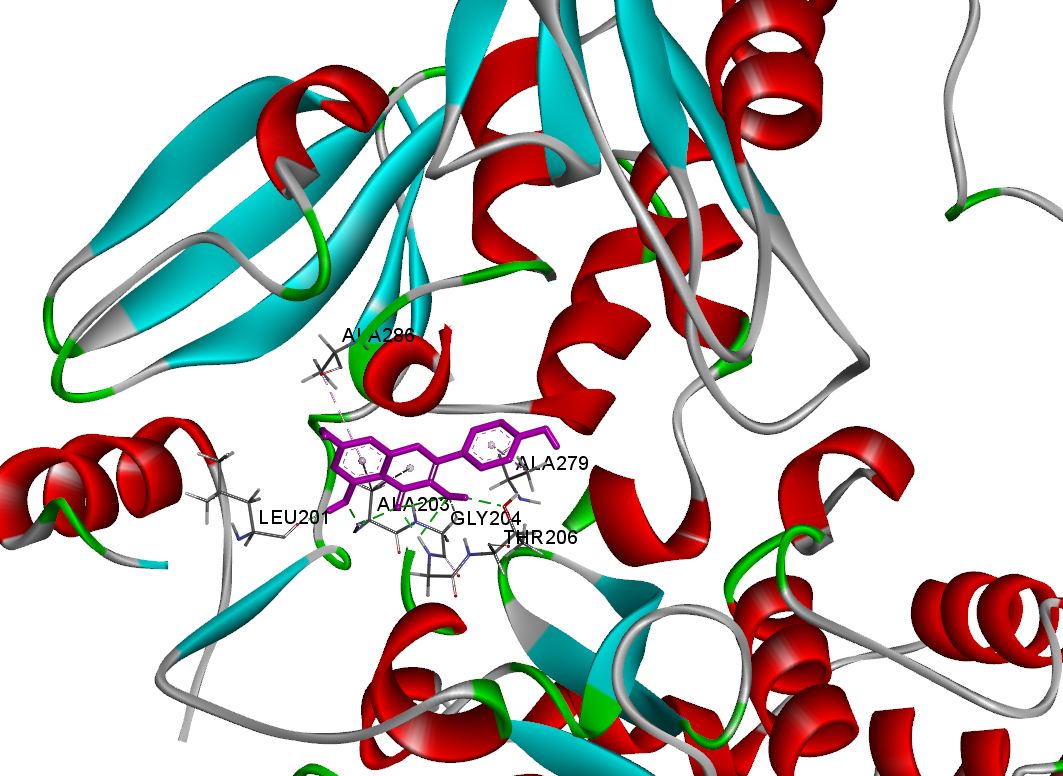 | 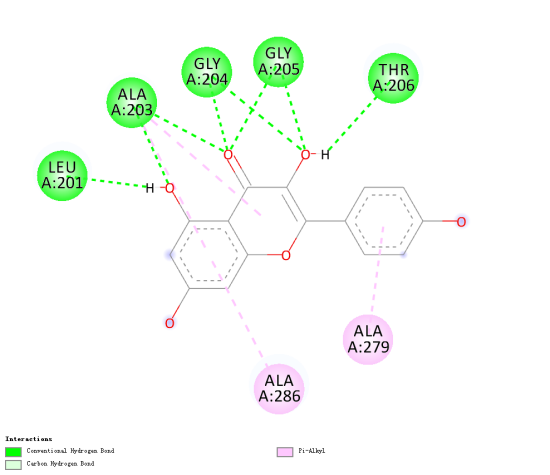 |
|  | | DPP4 | | 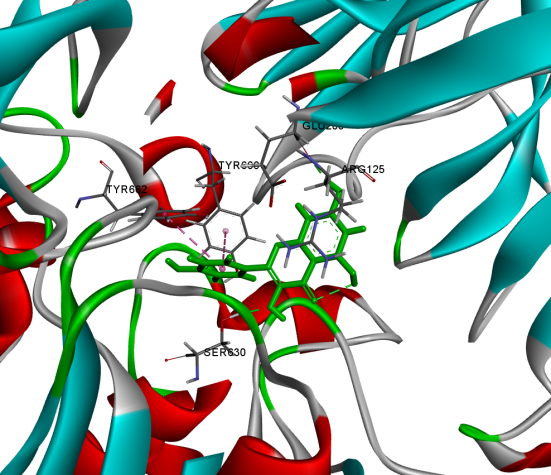 | 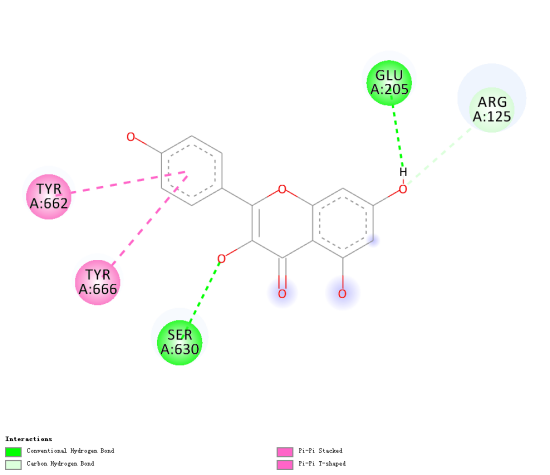 |
| β-sitosterol deposited substance | | MMP9 | | 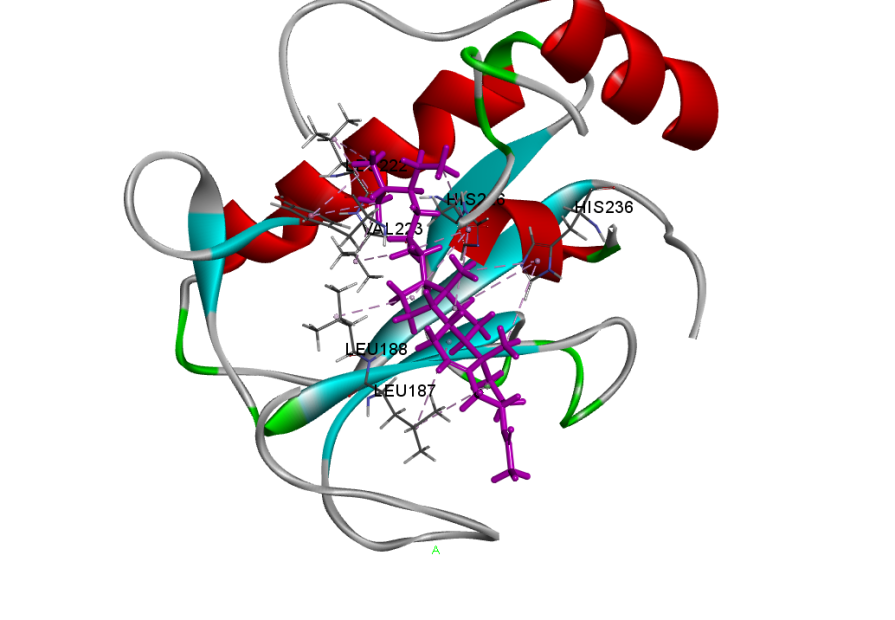 | 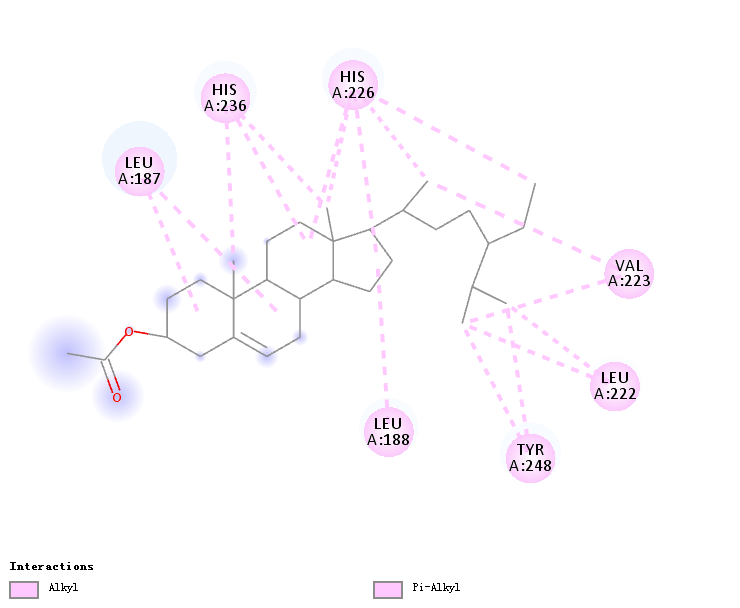 |
|  |  | PPARA | | 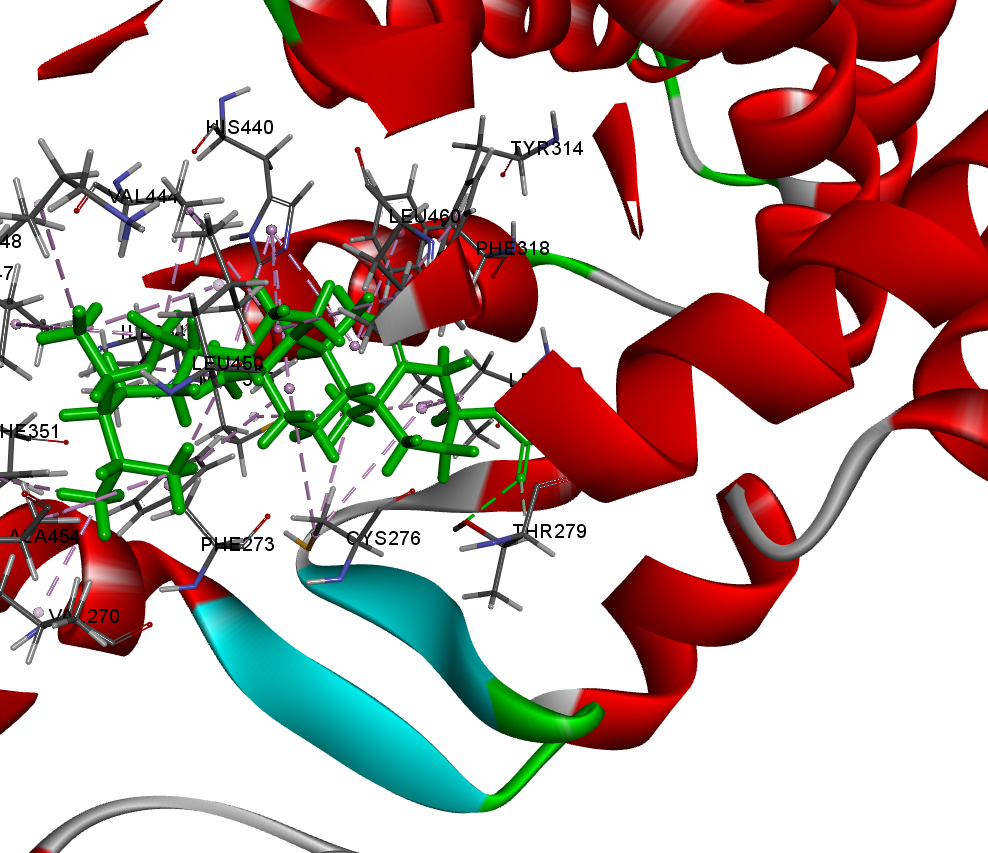 | 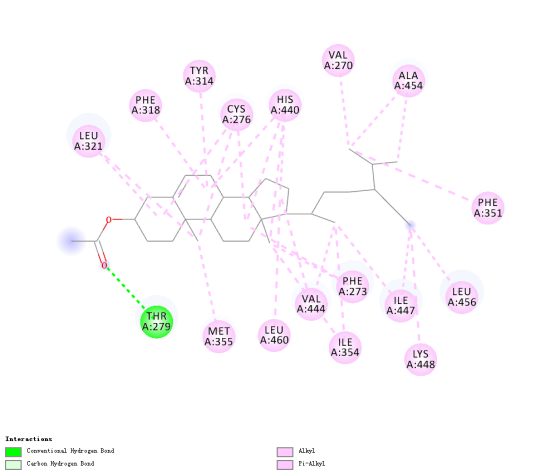 |
|  |  | HMOX1 | | 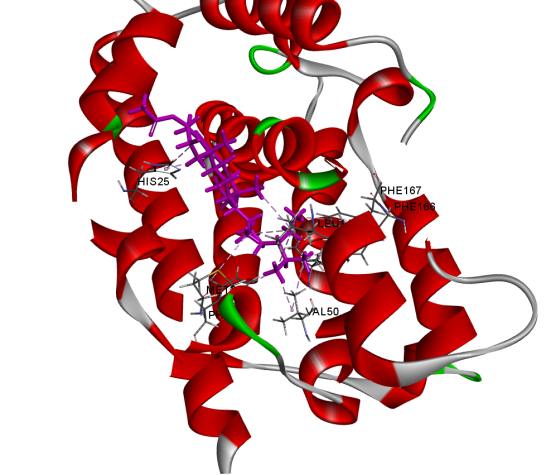 | 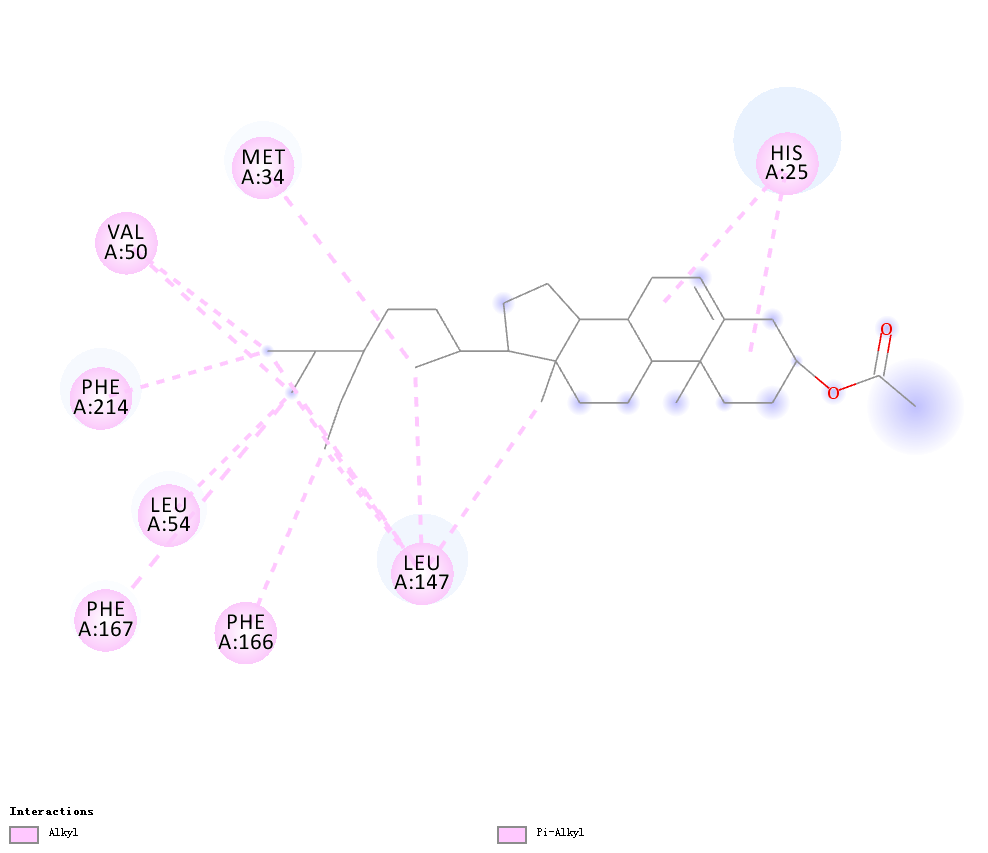 |
|  |  | OPRM1 | | 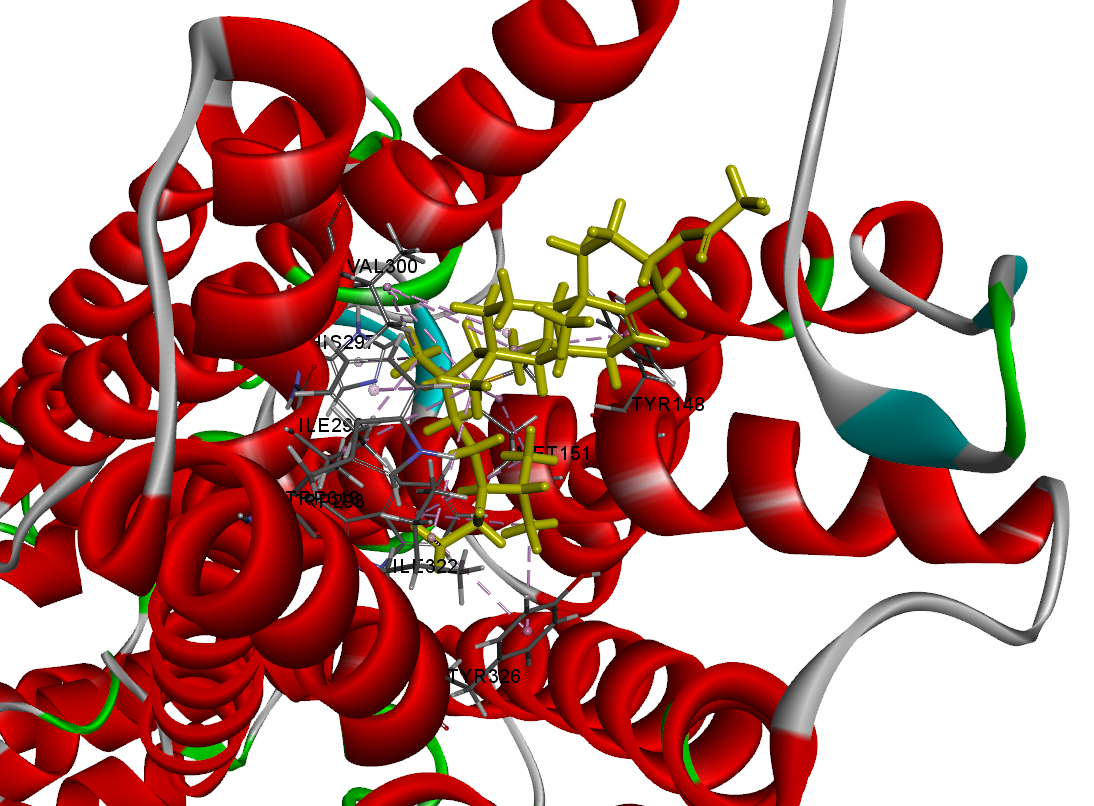 | 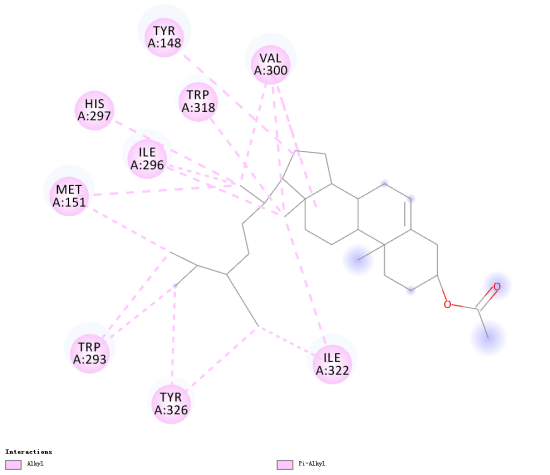 |
|  |  | NRC32 | | 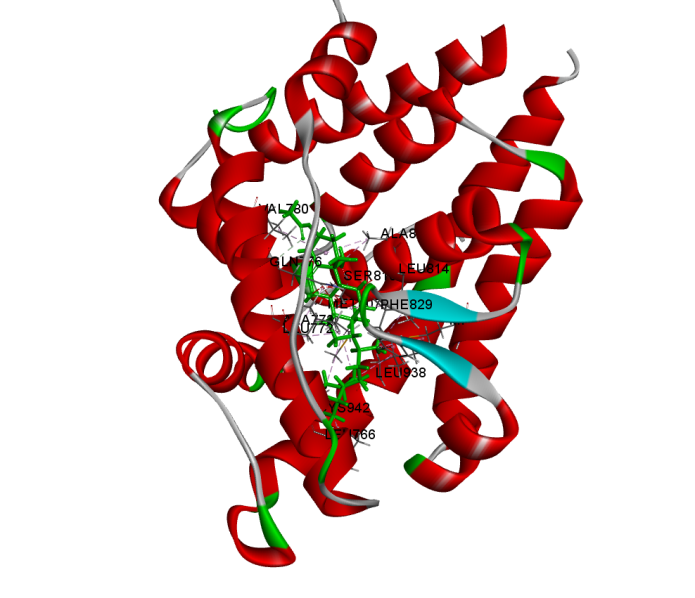 | 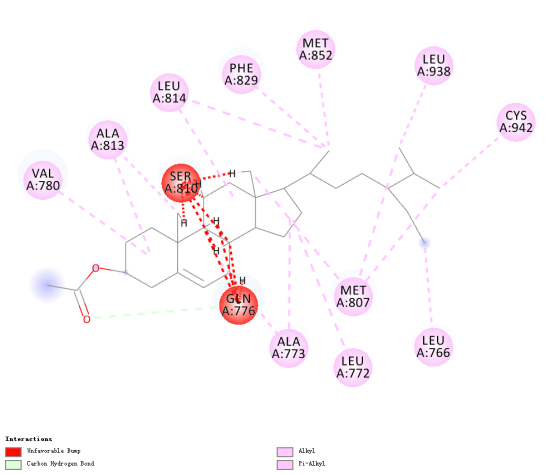 |
|  |  | ADRB2 | | 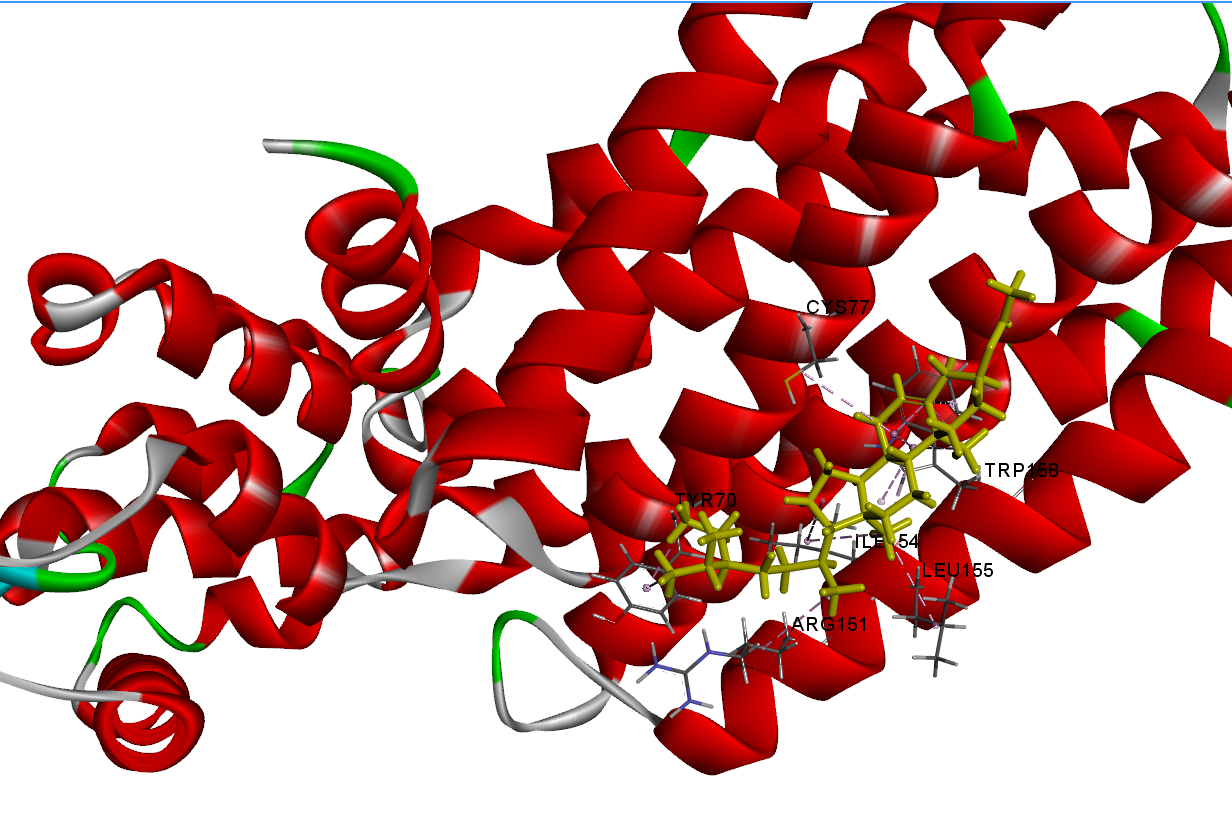 | 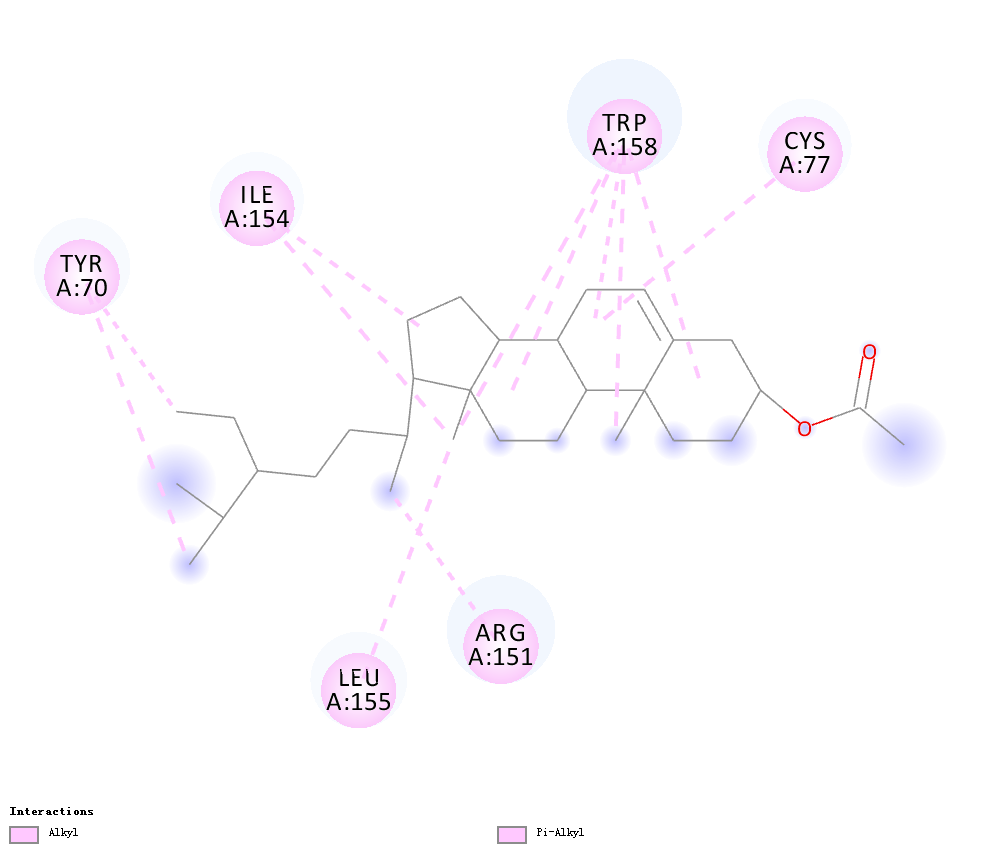 |
|  |  | XDH | | 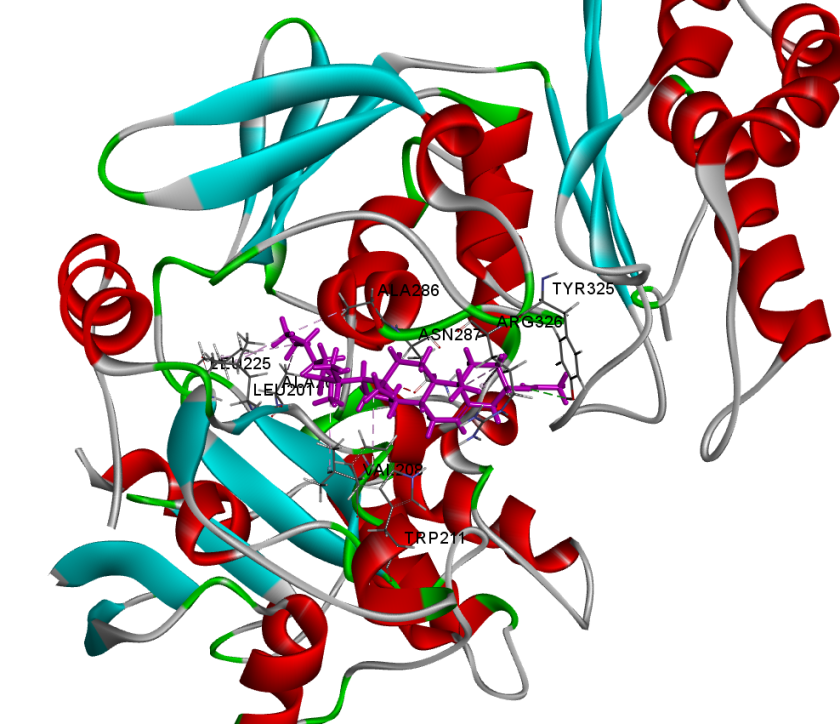 | 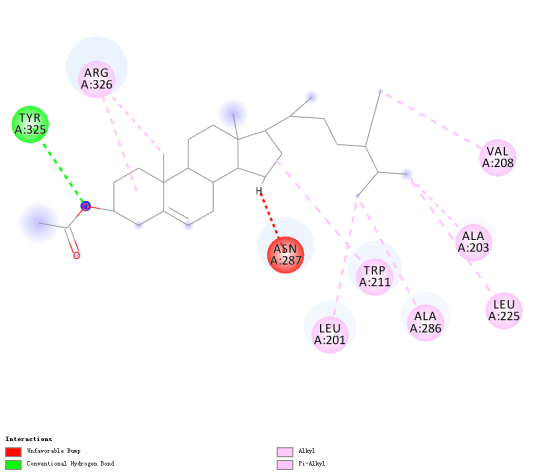 |
|  |  | DPP4 | | 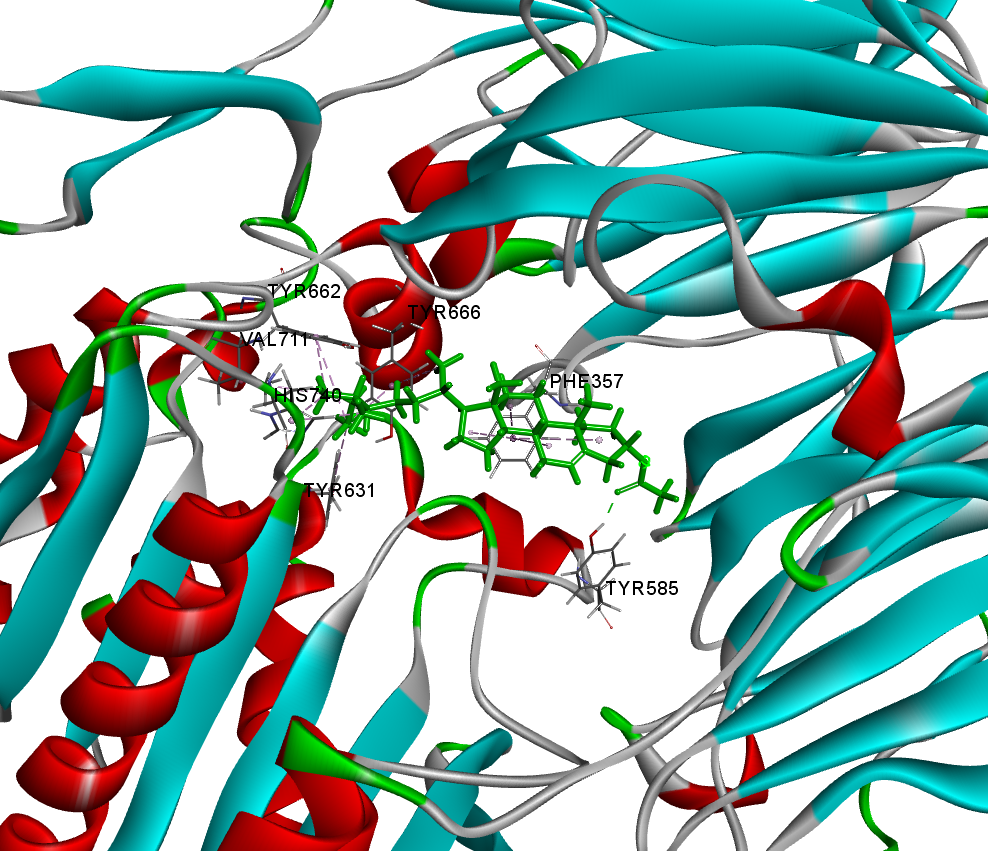 | 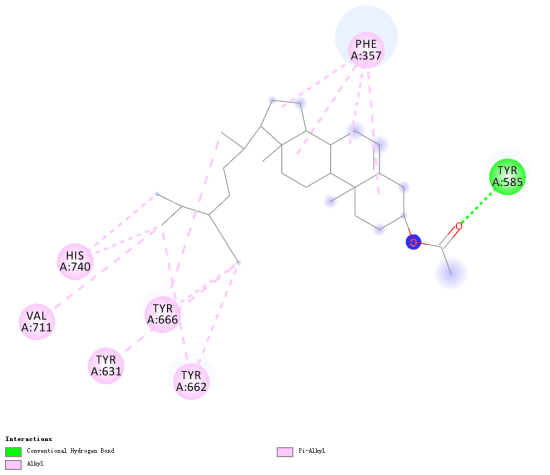 |
